# PDGFD: A Dual-Function Regulator That Maintains Myoblast Pool and Fuels Myogenic Differentiation

**DOI:** 10.3390/cimb48030322

**Published:** 2026-03-18

**Authors:** Hongzhen Cao, Jing Wang, Yunzhou Wang, Jingsen Huang, Wei Chen, Hui Tang, Junfeng Chen, Baosong Xing, Yongqing Zeng

**Affiliations:** 1 Shandong Provincial Key Laboratory for Livestock Germplasm Innovation & Utilization, College of Animal Science and Technology, Shandong Agricultural University, Tai’an 271018, China; 2 Henan Key Laboratory of Farm Animal Breeding and Nutritional Regulation, Henan Pig Breeding Engineering Research Centre, Institute of Animal Husbandry, Henan Academy of Agricultural Sciences, Zhengzhou 450002, China; 3 Department of Veterinary Medicine, Shandong Vocational Animal Science and Veterinary College, Weifang 261061, China

**Keywords:** *PDGFD*, functional validation, C2C12, PI3K-Akt signaling pathway, longissimus dorsi muscle

## Abstract

The role of platelet-derived growth factor D (*PDGFD*) in mesenchymal cells is well-established, but its specific function in skeletal muscle generation remains unknown. This study reveals for the first time PDGFD’s dual regulatory role in myogenesis: it acts both as a “guardian” maintaining the myoblast pool and as an “initiator” driving myogenic differentiation. Through single-cell RNA sequencing analysis of skeletal muscle from *Jiangquan Black pigs*, we identified *PDGFD* as a common candidate gene for both muscle and fat development. In the C2C12 cell model, *PDGFD* knockdown significantly inhibited cell proliferation and promoted apoptosis, while overexpression enhanced viability and inhibited apoptosis, indicating its critical role in maintaining myoprogenic precursor cell homeostasis. Further studies revealed that *PDGFD* interference downregulated key myogenic differentiation markers *MyoD* and *MyoG*, inhibiting differentiation. Its expression peaked during mid-differentiation (D5), suggesting temporal regulation of differentiation. Interestingly, although *PDGFD* primarily acts through the PI3K/Akt pathway downstream of *PDGFR-β*, *PDGFD* knockdown did not show significant synergistic effects with PI3K/Akt pathway activation in inhibiting differentiation. This suggests *PDGFD* may specifically regulate myogenic differentiation via an independent or parallel signaling axis. This study not only expands the known functions of *PDGFD* in muscle biology but also provides new insights into the mechanisms by which growth factors coordinate cell fate decisions.

## 1. Introduction

Platelet-derived growth factors (PDGFs) constitute a key family of signaling molecules that exert potent mitogenic and chemotactic effects on mesenchymal-derived cells such as fibroblasts, smooth muscle cells, and glial cells [1]. The PDGF family comprises four distinct polypeptide chains (*PDGFA*, *PDGFB*, *PDGFC*, and *PDGFD*), which assemble into five functional dimeric isoforms (PDGF-AA, -AB, -BB, -CC, and -DD). These ligands transmit signals through two receptor tyrosine kinases—*PDGFR-α* and *PDGFR-β*. Receptor binding to ligands triggers receptor dimerization and autophosphorylation, thereby activating downstream pathways—including the PI3K/AKT and MAPK/ERK pathways—that regulate cell proliferation, migration, survival, and differentiation [1,2,3,4,5].

Among these members, *PDGFD* was discovered relatively late and is unique in requiring proteolytic activation to achieve full biological activity [2,4]. Similarly to *PDGFB*, *PDGFD* primarily binds to and activates *PDGFR-ββ* homodimers [4]. Accumulating evidence indicates *PDGFD* participates in diverse physiological and pathological processes, including organ fibrosis, inflammation, angiogenesis, and tumorigenesis [6,7,8,9]. Its expression has been detected in multiple tissues, with notably elevated levels in the heart, kidney, pancreas, and adipose tissue, suggesting tissue-specific functions [2,4,10].

Skeletal muscle development and postnatal regeneration are highly dependent on the activity of muscle-specific stem cells—satellite cells. Upon activation, these cells proliferate, differentiate, and fuse to form new muscle fibers or repair damaged ones [11,12]. The mouse myoblast cell line C2C12 serves as a mature in vitro model for myogenesis. It can be induced to differentiate from myoblasts into multinucleated myotubes upon serum removal [12,13].

Although the functions of *PDGFA* and *PDGFB* in mesenchymal cells have been extensively studied, the role of *PDGFD*—particularly in skeletal muscle biology—remains poorly understood. Previous studies suggest it may participate in adipocyte maturation and homeostasis regulation [14], but its direct function in myogenic cells remains understudied. We utilized snRNA-seq data from *Jiangquan Black pig* muscle to identify conserved regulators of muscle development, as well as validated findings in the widely accepted mouse C2C12 myoblast model. However, a critical gap exists in our understanding of whether and how *PDGFD*, a factor implicated in adipogenesis, directly regulates the fundamental processes of skeletal myogenesis—specifically, the expansion of the myoblast pool and the subsequent execution of the differentiation program. Its potential role in coordinating the transition from proliferation to differentiation, a pivotal switch in muscle development, remains entirely elusive.

Based on its expression pattern and known functions, we hypothesized that *PDGFD* serves as a dual-function regulator in myogenesis, sustaining myoblast survival and proliferation during the growth phase, and subsequently promoting or permitting myogenic differentiation. To test this hypothesis, we aimed to systematically validate the functional role of *PDGFD* in skeletal muscle cells. Using the C2C12 cell model, we investigated the effects of *PDGFD* knockdown and overexpression on cell proliferation, apoptosis, cell cycle progression, and myogenic differentiation. Furthermore, by investigating its interaction with the canonical PI3K/AKT pathway, we sought to preliminarily determine whether PDGFD’s functions are mediated through this well-known survival axis or involve alternative signaling mechanisms. This study provides new insights into the role of *PDGFD* in myogenesis, laying the foundation for a deeper understanding of its significance in muscle physiology and related diseases.

## 2. Materials and Methods

### 2.1. snRNA-Seq Analysis

The experimental pigs were selected from *Jiangquan Black pigs* at a farm in Shandong Province. Based on average daily weight gain, four *Jiangquan Black pigs* with the highest and lowest average daily weight gains were chosen (Two heads in each group). Their longissimus dorsi muscles were extracted for single-nuclei RNA sequencing (snRNA-seq) using the 10× Genomics platform. The specific method involved lysing and disrupting tissues to isolate nuclei, followed by filtering using a flow cytometer before library construction and sequencing. Seurat was further employed to exclude cells with fewer than 24,000 unique molecular identifier (UMI) counts or those expressing fewer than 250 genes or more than 4900 genes. To address batch effects during clustering. Simultaneously, during sampling, we preserved the heart, liver, kidneys, spleen, lungs, adipose tissue, as well as the biceps brachii and trapezius muscles at −80 °C for subsequent gene expression analysis.

### 2.2. Cell Culture and Differentiation

The genetic screening was based on the results of single-cell nuclear sequencing in our laboratory, and the mouse muscle satellite cell line C2C12 (purchased from Wuhan Prosperity Life Sciences Co., Ltd., Wuhan, China) was used for the experiment. C2C12 cells between passages 8 and 15 were used for all experiments to ensure consistent differentiation potential. The medium of C2C12 muscle satellite cells was supplemented with 10% fetal bovine serum (FBS), 1% antibiotic mixture of penicillin, streptomycin, and gentamycin, and high-sugar Dulbecco’s Modified Eagle Medium (DMEM, Gibco, Carlsbad, CA, USA). Cells were cultured at 37 °C in a humidified incubator with 5% CO_2_. When C2C12 cells grew to 60–80% confluence, differentiation medium (2% horse serum (HS)) was added to induce them into undergoing differentiation, and samples were taken at different periods, with the start of induction recorded as D0, and cells differentiating D1, D3, D5, D7, and D9 were collected for subsequent experiments.

### 2.3. Analysis of PDGFD Sequence Information

The cDNA was synthesized by designing primers based on the sequence of the *PDGFD* gene in NCBI (XM_021062718.1), the primer sequences (see Table 1). The full-length of the gene was amplified with ApexHF HS DNA polymerase CL (AG12204) (Wuhan, China), after which the PCR products were gel-recovered (TIANGEN, DP209) (Beijing, China) and sequenced. The results obtained were subjected to blast comparison and then analyzed for conserved regions as well as evolutionary relationships of the genes using MEGA-X64.

### 2.4. Cellular Immunofluorescence

Cells were plated onto 6-well plate coverslips at 1 × 10^5^ cells/mL. After the specified culture period, the samples were processed for immunofluorescence. The procedure commenced with fixation in chilled 4% paraformaldehyde (30 min, 37 °C), followed by permeabilization using 0.1% Triton X-100 (Beijing, China) (40 min, RT). Non-specific sites were blocked with 5% FBS (1 h, 37 °C). The preparations were then incubated with the primary antibody (Proteintech, Cat# 14075-1-AP; 1:1000 dilution) (Wuhan, China) overnight at 4 °C and subsequently with Alexa Fluor 647-tagged secondary antibody (1:1000, 1 h, 37 °C) in the dark. Nuclei were visualized by DAPI staining (10 min, 37 °C). Between each step, extensive rinsing with PBS was performed. The images were acquired using an Andor confocal live-cell imaging system (Dragonfly, Hertfordshire, UK).

### 2.5. Plasmid Construction and Cell Transfection

The sequence of the interfering fragment was designed according to its CDS region (see Table 2), and the full-length cDNA of *PDGFD* was obtained by PCR using PrimeSTAR Max DNA Polymerase (Takara, Dalian, China). The PCR products were subjected to gel electrophoresis and gel recovery sequencing, and the sequences were aligned with those in NCBI to ensure correctness. The PCR products and the pEGFP-N1 plasmid (Clontech, Mountain View, CA, USA) were digested with FastDigest HindIII and BamHI (Chengdu, China), and then the *PDGFD* fragment was ligated into the linearized pEGFP-N1 vector with T4 DNA ligase (Chengdu, China) to generate the PDGFD-pEGFP-N1 plasmid, which was verified by sequencing. For transformation, 10 μL of the recombinant plasmid was added to 50 μL of DH5α competent cells (Tiangen Biotech, Beijing, China), following the manufacturer’s instructions. Positive clones were selected on LB solid medium containing 50 μg/mL kanamycin and verified by colony PCR. Then, the validated recombinant plasmids were extracted using the Tian Gen Plasmid Extraction Kit (Tiangen Biotech, Beijing, China) for subsequent experiments. For transfection, cells were seeded into 6-well plates (Corning, Corning, NY, USA) at a density of 1 × 10^5^ cells per well one day prior to transfection. When the cell confluence reached 60–80%, the culture medium was replaced with serum-free Opti-MEM (Gibco). Transfection was performed using Hieff Trans Liposomal Transfection Reagent (Yeasen, Shanghai, China) according to the manufacturer’s protocol. Specifically, 4 μL of siRNA (20 μM) or 5 μg of overexpression vector was diluted in 250 μL Opti-MEM, mixed gently with the transfection reagent, and added to the cells. After 6 h of incubation, the medium was replaced with fresh complete growth medium. Each experiment was performed with at least three independent biological replicates (n = 3), defined as cells harvested from separate transfections performed on different days using cells from different passages. Non-targeting siRNA (si-NC) and empty pEGFP-N1 vector (Vector) were used as negative controls in all relevant experiments. Transfection efficiency was confirmed by Western blot analysis of PDGFD protein levels.

### 2.6. Cell Proliferation Assay

Cell proliferation activity was assessed using the CCK-8 assay kit (Yeasen, Shanghai, China). Log-phase cells were seeded at 100 μL per well into a 96-well plate, with six replicate wells per group. An additional six wells contained an equal volume of complete medium as a blank control. After 12 h of incubation, cells were transfected with the interference fragment and overexpression vector. Cell viability was assessed at 12, 24, 36, and 48 h post-transfection: 10 μL of CCK-8 working solution was added to each well. Plates were incubated in the incubator, protected from light, for 2 h, followed by measurement of absorbance at 450 nm using a microplate reader.

To observe cell proliferation using the EdU-488 assay kit (Shanghai, China), pre-place cell coverslips in a 6-well plate, dispensing 200 μL of cell suspension onto each coverslip. After 1 h of culture to allow cell attachment, slowly add 1.5 mL of growth medium to each well. Proceed with transfection once cell density reaches 70–80%. Twenty-four hours post-transfection, add the EdU-488 reagent (Shanghai, China) and continue incubation for 2 h. All subsequent procedures must be strictly adhered to in accordance with the kit instructions.

### 2.7. Cell Cycle and Apoptosis Assay

Following transfection and expansion to the required cell density (1 × 10^6^ cells), cell cycle and apoptosis assays were conducted separately. Cell cycle analysis: Processed using the Cell Cycle and Apoptosis Detection Kit (Beyotime, C1052) (Shanghai, China). Following cell collection, strictly adhere to the protocol instructions, employing an FSC/SSC-based gating strategy for flow cytometric analysis. Apoptosis Analysis: Assessed using the Annexin V-FITC Apoptosis Detection Kit (Beyotime, C1062M) (Shanghai, China), with all steps performed according to the manufacturer’s protocol. Upon completion of the aforementioned experiments, statistical analysis of the data was conducted using FlowJo software (version 10.8.1).

### 2.8. Real-Time Fluorescent Quantitative PCR Analysis

Total cellular RNA was extracted using an RNA extraction kit (TIANGEN, RNA simple Total RNA Extraction Kit), followed by cDNA synthesis with a reverse transcription kit (Evo M-MLV Reverse Transcription Premix Kit, Accurate Biotechnology) (Wuhan, China). The reverse transcription reaction employed a two-step method: initial incubation at 42 °C for 2 min, followed by incubation at 37 °C for 15 min, then at 85 °C for 5 s, and finally storing it at 4 °C. The reaction system contained 1 µg of total RNA in a total volume of 20 µL.

qPCR amplification was performed using the cDNA as a template, employing the SYBR^®^ Green Pro Taq HS Pre-mixed qPCR Kit (Accurate Biotechnology) for gene expression analysis. The reaction system comprised 10 µL SYBR Premix Ex Taq (2×) and forward/reverse primers (2 µM, 0.4 µL each). All reactions were performed in triplicate. β-actin served as the housekeeping gene, and relative gene expression was calculated using the 2^−ΔΔCt^ method, where ΔCt = Ct target gene − Ct housekeeping gene. Species-specific primers were employed for both porcine and murine samples; primer sequences are detailed in Table 3. All experimental procedures were conducted on ice.

### 2.9. Western Blot

Following treatment, allow cells to grow to the designated time point. Add RIPA lysis buffer containing PMSF (Biyun Tian, P0013B, Shanghai, China) to lyse the cells. Collect the lysate into a 1.5 mL centrifuge tube, mix thoroughly, then centrifuge at 4 °C and 12,000 rpm for 5 min. Remove the supernatant. Determine protein concentration using the BCA Protein Concentration Assay Kit (Beyotime) (Shanghai, China). Dilute all protein samples to an identical concentration with RIPA buffer, add 5× SDS-PAGE loading buffer in proportion, heat in a 100 °C water bath for 10 min, then store temporarily at −20 °C. Perform protein electrophoresis using elife pre-made gel, running under the required voltage conditions. Following electrophoresis, transfer proteins to a PVDF membrane using Xinsaimai Rapid Blotting Transfer Solution (taking care to avoid bubbles). After transfer, block at 37 °C for 30 min. Subsequently, add the primary antibody diluted at a ratio of 1:1000 and incubate overnight at 4 °C. After recovering the primary antibody, wash with 1× TBST. Add the secondary antibody diluted 1:1000 and incubate at room temperature for 2 h. Finally, wash with TBST and perform chemiluminescent imaging using ECL chemiluminescent substrate (Biyun Tian) to detect protein bands. All primary antibodies used in these experiments were purchased from Proteintech as follows: Beta-Actin Recombinant antibody (8115-1-RR), MYOD1 Rabbit PolyAb (18943-1-AP), MYOG Polyclonal antibody (26762-1-AP), and MEF2C Polyclonal antibody (10056-1-AP). Goat Anti-Rabbit IgG, HRP Conjugated (CW0103S). Pre-stained Protein Marker 10-180KD (Cat No.: P1018).

### 2.10. Pathway Activator

After transfection (designated as Day 0), to activate the PI3K/Akt signaling pathway, the culture medium was replaced with a fresh differentiation medium containing 20 μmol/L SC79 (MedChemExpress, Monmouth Junction, NJ, USA), a specific Akt activator. This concentration was selected based on previous studies showing effective pathway activation in C2C12 myoblasts. An equivalent volume of dimethyl sulfoxide (DMSO, Sigma-Aldrich, St. Louis, MO, USA), the solvent for SC79, was added to the control groups to account for any potential solvent effects. The medium, along with the activator or DMSO, was refreshed every 24 h throughout the subsequent differentiation period until the cells were harvested for analysis.

### 2.11. Statistical Analysis

Quantitative analysis of protein band gray values was performed using ImageJ 1.5x software. All experimental data underwent statistical processing using IBM SPSS Statistics 25 software. Comparisons between two groups were performed using the independent-samples *t*-test, while comparisons among multiple groups employed one-way analysis of variance (ANOVA). Statistical results are presented as mean ± standard error of the mean (SEM), with statistical graphs generated using GraphPad Prism 8.0 software. Differences were considered statistically significant at * *p* < 0.05; ** *p* < 0.01; *** *p* < 0.001., respectively.

## 3. Results

### 3.1. Analysis of Sequencing Results

**Figure 1:**
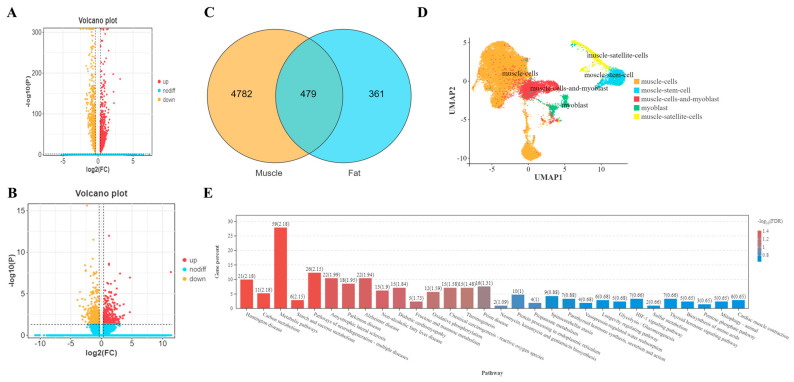
Analysis of sequencing results. (**A**) Analysis of differences between muscle cell-related clusters; (**B**) analysis of differences between adipocyte-related clusters (the dashed line parallel to the X-axis represents the *p* = 0.05 threshold line, while the dashed line parallel to the Y-axis represents the |log_2_FC| = 1 threshold line). (**C**) Venn plots of gene set analysis of different cell types; (**D**) UMAP Plot of Muscle-Related Subgroup Classification Information; (**E**) bar graph of KEGG enrichment of overlapping target genes.

To identify novel regulators of muscle development, we performed snRNA-seq on the longissimus dorsi muscle of Jiangquan black pigs exhibiting high (H-ADG) and low (L-ADG) average daily gain. Comparative analysis of muscle-associated cell populations between H-ADG and L-ADG groups identified 775 upregulated and 827 downregulated genes (Figure 1A). Simultaneously, analysis of adipocyte populations within the same tissues revealed 418 upregulated and 422 downregulated genes (Figure 1B). A Venn diagram intersection of these differentially expressed gene sets identified 479 common genes (Figure 1C). We generated UMAP plots for muscle-related subpopulations for visualization (Figure 1D). KEGG pathway enrichment analysis of these 479 shared genes highlighted significant involvement in metabolic pathways and the PI3K-Akt signaling pathway (Figure 1E). Based on its known functions in mesenchymal cell biology and the unique pattern of differential expression observed in both muscle and fat cell populations within our sequencing data (suggesting potential regulatory roles across cell types), we selected *PDGFD* for subsequent in-depth functional validation.

### 3.2. Analysis of PDGFD Gene Sequence Information

**Figure 2:**
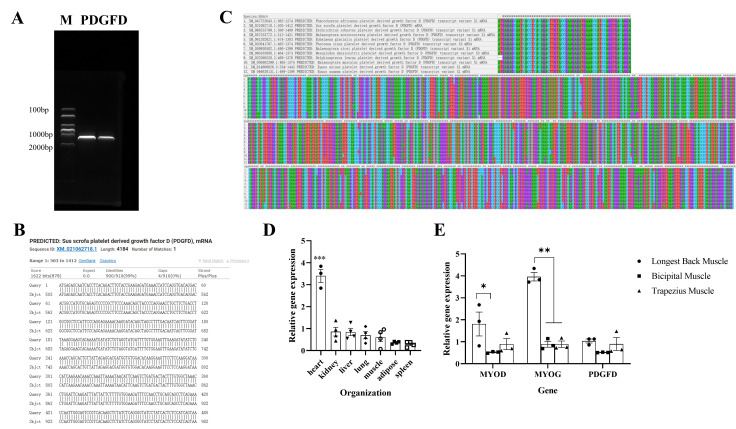
Sequence information analysis. (**A**) Nucleic acid gel electrophoresis; (**B**) comparison results of the sequences on NCBI (screenshot); (**C**) conserved domain analysis of MEGA-X sequences; (**D**) tissue expression profiles; (**E**) expression of the genes in different muscle tissues (for (**D**,**E**), the different symbols on the bar charts represent biological replicates) (n = 3, * *p* < 0.05, ** *p* < 0.01, *** *p* < 0.001).

In this study, we successfully de-amplified the CDs of the *PDGFD* gene (Figure 2A,B), and subsequently downloaded 12 *PDGFD* sequences of different species including the target gene from NCBI, and by comparison, we found that the sequences of the *PDGFD* gene were still relatively conserved among the species (Figure 2C). On analysis of its amino acids, the isoelectric point was found to be roughly 9.75. After identifying the gene, the expression profiles of the different organs as well as the different tissues of the gene were briefly analyzed by using the pre-laboratory sample storage, and it was found that the expression of the gene was highly significantly higher in the heart than in the other organs (*p* < 0.001) (Figure 2D), and the gene was more abundantly expressed in the dorsal longest muscle than in the biceps brachii muscle and the obliques, but there was no significant difference (*p* > 0.05) (Figure 2E).

### 3.3. Detection of the Effect of Induced Differentiation in C2C12 Cells

**Figure 3:**
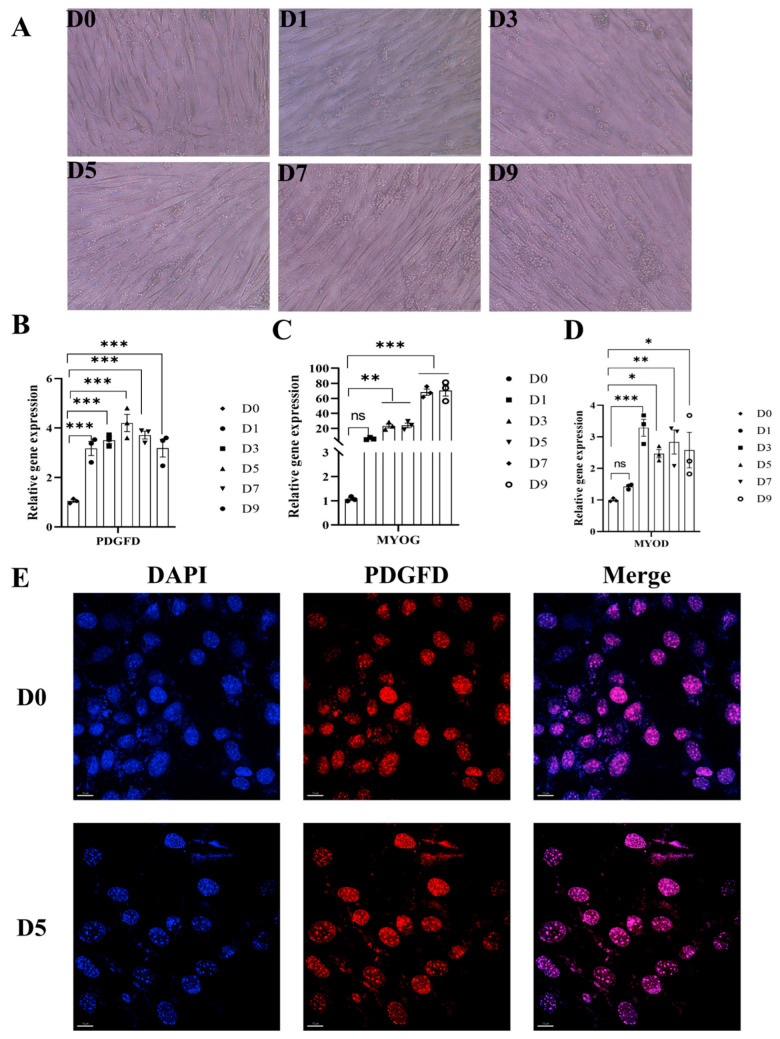
Detection of the effect of induced differentiation of C2C12 cells. (**A**) Myotube fusion during C2C12 differentiation (40X), scale bar = 500 px; (**B**) expression of *PDGFD* gene at different differentiation days (n = 3); (**C**) expression of *MYOG* gene at different differentiation days (n = 3); (**D**) expression of *MYOD* gene at different differentiation days (n = 3) (for Figures (**B**–**D**), the different symbols on the bar charts represent biological replicates); (**E**) functional localization of the *PDGFD* gene (blue fluorescence for the nucleus (DAPI), red fluorescence for localization of the PDGFD protein, scale bar = 15 μm, 63X) (* *p* < 0.05, ** *p* < 0.01, *** *p* < 0.001).

Fluorescent quantitative PCR analysis revealed that *PDGFD* gene expression peaked on day 5 of differentiation (D5), with highly significant differences observed at all differentiation time points compared to D0 (*p* < 0.001) (Figure 3B). The expression of myogenic marker genes was upregulated as expected during differentiation: *MYOG* and *MYOD* expression at D3, D5, D7, and D9 was significantly higher than at D0 (*p* < 0.01) (Figure 3C,D); and the *MYOD* gene at D1 was not significantly different (*p* > 0.05) compared to that at D0, but all other days were significantly different (*p* < 0.05) compared to D0 (Figure 3D). Furthermore, immunofluorescence staining using anti-PDGFD antibodies revealed that this protein is primarily localized within the cell nucleus in C2C12 cells (Figure 3E) (for more complete image details, see Supplementary Material S1). Due to the lack of negative controls following gene knockout or knockdown, this localization result should be interpreted with caution, as it may reflect the processing of its precursor protein prior to secretion.

### 3.4. Screening of Overexpression Vectors and Interfering Fragments

**Figure 4:**
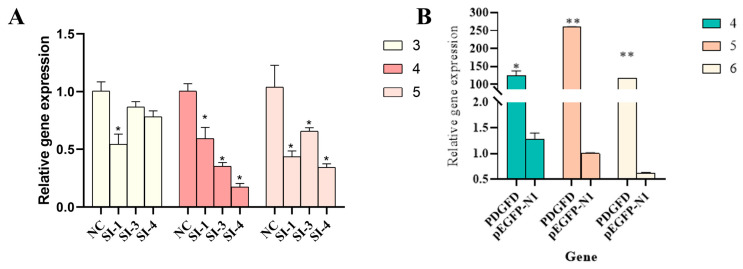
Screening of overexpression vectors and interfering fragments. (**A**) Effect of different concentrations of different interfering fragments on target genes; (**B**) effect of different concentrations of overexpression on target genes (n = 3, * *p* < 0.05, ** *p* < 0.01).

Quantification of different concentrations of the overexpression vectors revealed that there were significant differences between the 4 μg, 5 μg, and 6 μg overexpression vectors compared with the null load (*p* < 0.05), and the differences reached a highly significant level (*p* < 0.01), especially when the concentrations of the vectors were 5 μg or 6 μg (Figure 4B). There were also significant differences (*p* < 0.05) between different interference fragments compared with NC (Figure 4A). Ultimately, based on its remarkable gene-silencing efficiency, we selected interference fragment 4 (SI-4, 4 μL per well) for subsequent experiments; the overexpression vector was administered at a concentration of 5 μg per well. It should be noted that this section only performed preliminary screening at the mRNA level. The efficiency of interference and overexpression at the protein level will be validated in subsequent functional experiments.

### 3.5. Effect of PDGFD on the Proliferation of C2C12 Cells

**Figure 5:**
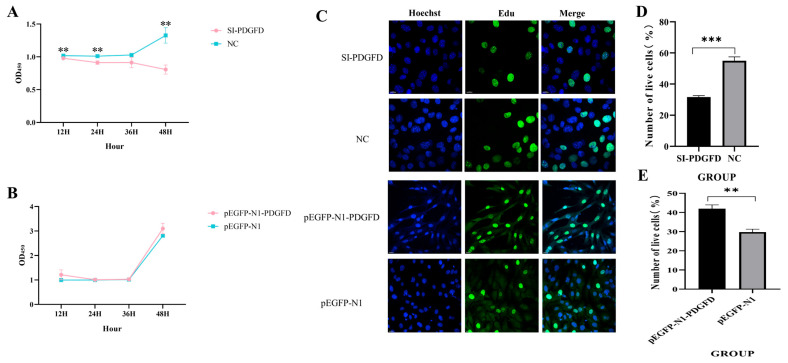
Effect of *PDGFD* gene on cell proliferation. (**A**) CCK-8 detects the effect of interfering *PDGFD* gene on cell proliferation ability (n = 6); (**B**) CCK-8 detects the effect of overexpressing *PDGFD* gene on cell proliferation ability (n = 6); (**C**) Edu detects the effect of *PDGFD* gene on cell proliferation, scale bar = 20 μm; (**D**) quantification of the effect of Edu detection on cell proliferation following *PDGFD* gene interference; (**E**) quantification of the effect of Edu detection on cell proliferation following *PDGFD* gene overexpression (** *p* < 0.01, *** *p* < 0.001).

Following transfection, this study investigated the effects of the target gene on cell proliferation and viability through two approaches: Edu-488 and CCK-8 assays. Results revealed that *PDGFD* interference significantly reduced cell viability at 12, 24, and 48 h post-transfection compared to the NC group (*p* < 0.01) (Figure 5A). Overexpression of PDGFD showed an increasing trend in cell viability, although the difference was not statistically significant (Figure 5B). As a complementary method, the Edu-488 test results further corroborate the aforementioned conclusions (Figure 5C) (for more complete image details, see Supplementary Material S2). In summary, *PDGFD* gene interference inhibits cell proliferation and viability, while *PDGFD* gene overexpression promotes cell proliferation and viability.

### 3.6. Effects of PDGFD on Cell Cycle and Apoptosis

**Figure 6:**
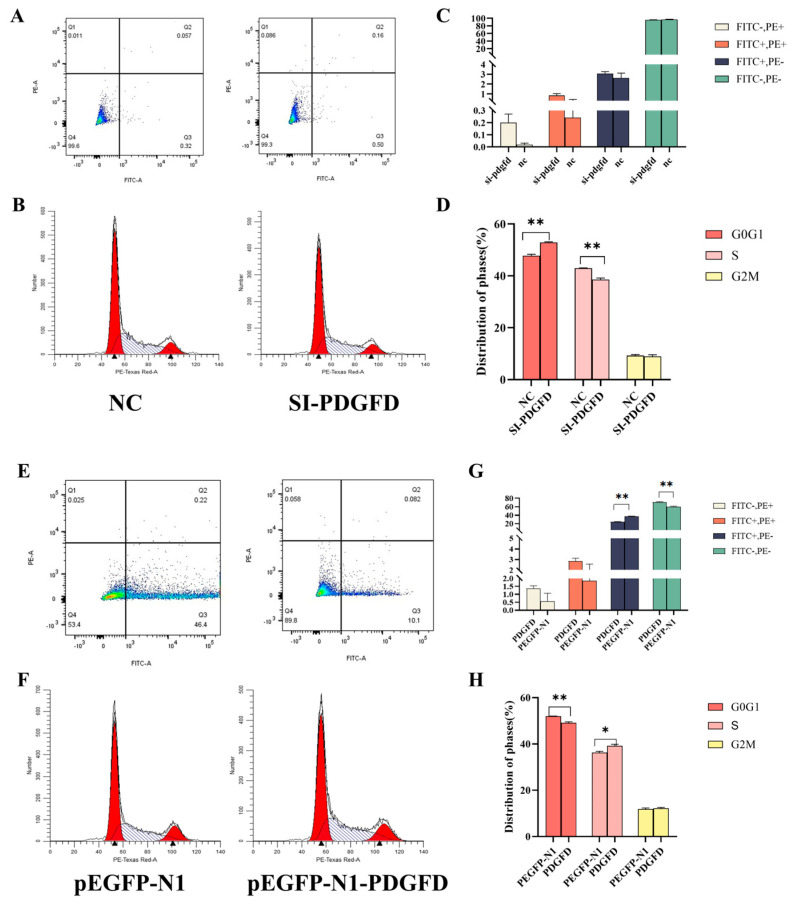
Flow assay for cell cycle and apoptosis. (**A**,**C**) Effect of interference on apoptosis of C2C12 cells; (**B**,**D**) effect of interference on C2C12 cell cycle; (**E**,**G**): effect of overexpression on apoptosis of C2C12 cells; (**F**,**H**) effect of overexpression on C2C12 cell cycle (n = 3, * *p* < 0.05, ** *p* < 0.01).

The interference fragment and overexpression vector were co-transfected into cells. C2C12 cells were cultured for approximately 24 hours before staining. Cell cycle changes and apoptosis (Annexin V-FITC/PI double staining) were assessed separately. Flow cytometry results showed that 24 hours after transfection with the interference fragment, C2C12 cells exhibited a trend toward increased apoptosis, but the difference was not statistically significant (*p* > 0.05) (Figure 6A,C). The proportion of cells in the GO/G1 phase was significantly higher in the interference group than in the NC group (*p* < 0.01), while the proportion of cells in the S phase was significantly lower in the interference group than in the NC group (*p* < 0.01). (Figure 6B,D). The overexpression vector significantly inhibited apoptosis (*p* < 0.01) (Figure 6E,G). The empty vector group exhibited a significantly higher proportion of cells in the G0/G1 phase than the overexpression group (*p* < 0.01), as well as a significantly lower proportion of cells in the S phase than the overexpression group (*p* < 0.05) (Figure 6F,H). These results indicate that overexpression inhibits apoptosis, interferes with apoptosis promotion, and has no significant effect on the cell cycle. For information on the apoptosis control group, see Supplementary Material S3.

### 3.7. Effect of PDGFD on Myogenic Differentiation of C2C12 Cells

#### 3.7.1. Effect of Interfering with PDGFD Gene on Myogenic Differentiation of C2C12 Cells

**Figure 7:**
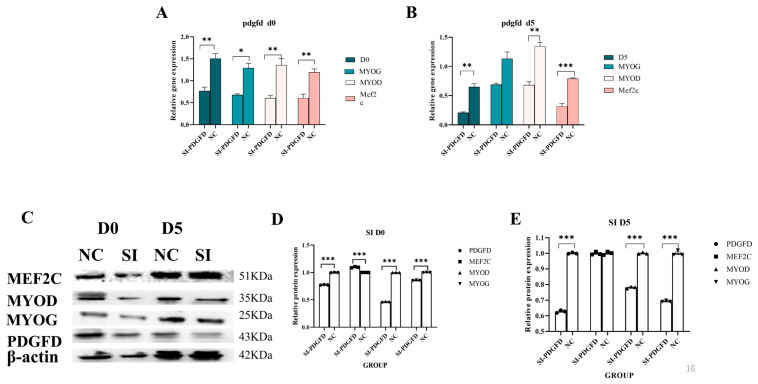
Effect of interfering with *PDGFD* gene on myogenic differentiation of C2C12 cells. (**A**) Interfering with the expression of D0-related genes of *PDGFD* gene; (**B**) interfering with the expression of D5-related genes of *PDGFD* gene; (**C**–**E**) Western blot to detect the expression level of the proteins of the D0 and D5-related genes, as well as analyze the intensity of the bands by using image J1.5x analysis software (* *p* < 0.05, ** *p* < 0.01, *** *p* < 0.001).

In this study, the interfering fragments and NC controls were co-transfected into C2C12 cells. RNA and protein samples were collected on days D0 and D5, respectively, and their effects were investigated using quantitative real-time PCR and Western blot techniques. Quantitative results revealed that the interference agents inhibited cell differentiation at both D0 and D5. Specifically, at D0, the differentiation marker genes *MYOD* and *MEF2C* showed significantly higher expression levels in the NC group compared to the interference group (*p* < 0.01), while *MYOG* expression was significantly lower in the infection group than in the interference group (*p* < 0.05) (Figure 7A). At D5, the expression levels of *MYOD* and *MEF2C* genes in the NC group were significantly higher than those in the interference group (*p* < 0.01) (Figure 7B). However, Western blot results revealed that at D0 and D5, the protein expression levels of PDGFD, MYOD, and MYOG in the interference group (SI) were significantly lower than those in the NC group (*p* < 0.001), while the protein levels of MEF2C were not significantly affected (*p* > 0.05). (Figure 7C–E).

#### 3.7.2. Effect of Overexpression of PDGFD Gene on Myogenic Differentiation of C2C12 Cells

**Figure 8:**
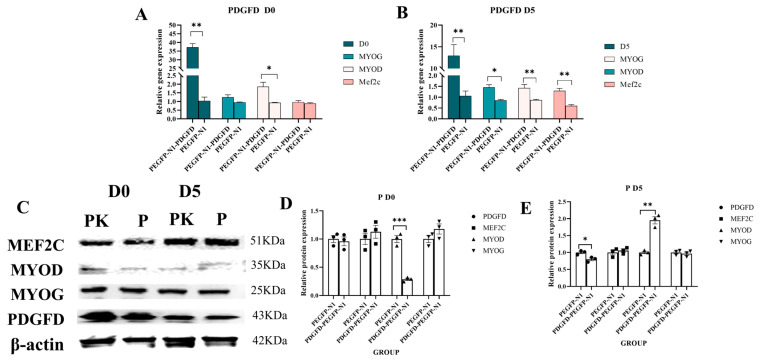
Effect of overexpression of *PDGFD* gene on myogenic differentiation of C2C12 cells. (**A**) expression of D0-related gene of overexpression of *PDGFD* gene; (**B**) expression of D5-related gene of overexpression of *PDGFD* gene; (**C**–**E**) Western blot to detect the expression level of protein of D0 and D5-related genes, as well as analyze the image J 1.5x analysis software (* *p* < 0.05, ** *p* < 0.01, *** *p* < 0.001).

To investigate the effect of *PDGFD* gain-of-function on myogenic differentiation, we transfected C2C12 cells with the pEGFP-N1-PDGFD overexpression vector. qPCR analysis performed at differentiation days 0 and 5 revealed that *PDGFD* overexpression tended to increase mRNA levels of the myogenic markers *MYOG* and *MYOD*. For example, at D0, the differentiation marker gene *MYOD* showed significantly higher expression in the overexpression group compared to the empty vector group (*p* < 0.05; Figure 8A). At differentiation day 5 (D5), the expression levels of the *MYOD* and *MEF2C* genes in the empty vector group were significantly lower than those in the overexpression group (*p* < 0.01), while the expression level of the MYOG gene in the overexpression group was significantly higher than that in the empty vector group (*p* < 0.05; Figure 8B). This trend was corroborated at the protein level by Western blot analysis (Figure 8C–E).

### 3.8. Combined Effect of PDGFD Knockdown and PI3K/AKT Pathway Activation on Myogenic Differentiation

**Figure 9:**
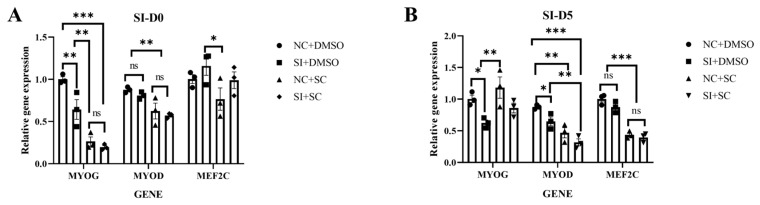
Combined effect of PDGFD knockdown and PI3K/AKT pathway activation on C2C12 cell differentiation. (**A**) Effect of interfering with *PDGFD* gene while adding PI3K/AKT pathway activator D0 on C2C12 cell differentiation; (**B**) effect of interfering with *PDGFD* gene while adding PI3K/AKT pathway activator D5 on C2C12 cell differentiation (n = 3, ns: *p* > 0.05, * *p* < 0.05, ** *p* < 0.01, *** *p* < 0.001).

Given that *PDGFD* primarily signals through PDGFR-β, which in turn activates the PI3K/Akt pathway, we investigated whether PDGFD’s regulation of myogenic differentiation depends on this pathway. As shown in Figure 9, treatment with the Akt activator SC79 alone (NC+SC79 group) significantly suppressed the expression of myogenic markers *MYOG* and *MYOD* at both D0 and D5 (*p* < 0.001). In *PDGFD*-knockdown cells (SI group), expression of these markers was also suppressed. However, combining *PDGFD* knockdown with SC79 treatment (SI+SC79 group) did not result in a further significant decrease in *MYOG* and *MYOD* expression levels compared to SC79 treatment alone (NC+SC79 group). This indicates that under the experimental conditions, no significant additive or synergistic effects were observed between the inhibitory effect of *PDGFD* knockdown on differentiation and the potent suppression caused by sustained activation of the PI3K/AKT pathway.

## 4. Discussion

This study provides the first functional evidence that *PDGFD*, a member of the platelet-derived growth factor family with known roles in fibrosis and adipogenesis, plays a crucial and multifaceted role in skeletal myogenesis. Using the C2C12 myoblast model, we demonstrate that *PDGFD* acts not merely as a mitogen, but as a dual-function regulator: it is essential for maintaining the proliferative myoblast pool and serves as a positive driver of terminal myogenic differentiation. Furthermore, our data intriguingly suggest that these two functions may be orchestrated through distinct, or at least partially separable, signaling modalities.

### 4.1. PDGFD as a Guardian of the Myoblast Pool: Promoting Survival over Proliferation

Our results firmly establish *PDGFD* as a key survival and pro-proliferation factor for myoblasts. The significant reduction in cell viability upon *PDGFD* knockdown, coupled with the increased apoptotic rate, aligns with the well-established role of the PDGF family as potent survival factors for mesenchymal cells [5,14,15]. However, the lack of a significant change in cell cycle distribution upon *PDGFD* manipulation is particularly insightful. This indicates that PDGFD’s primary contribution to the expanding myoblast population is not through forcefully pushing cells through the cell cycle, but rather through creating a permissive microenvironment by suppressing apoptosis. This “guardian” function is critical for maintaining an adequate reservoir of myogenic precursors, a prerequisite for effective muscle growth and regeneration.

### 4.2. PDGFD as an Initiator of Myogenic Differentiation: A Novel Role Beyond Mitogenesis

More importantly, we uncover a previously unrecognized and direct role for *PDGFD* in promoting myogenic differentiation. Knockdown of PDGFD led to a consistent downregulation of the master regulators MyoD and MyoG at both mRNA and protein levels. Given that MyoD commits cells to the myogenic lineage and MyoG executes terminal differentiation and fusion [16], their suppression strongly indicates a compromised differentiation program. The dynamic expression pattern of *PDGFD* during differentiation—peaking at mid-stage (D5)—further supports its active participation in the differentiation process rather than being a passive bystander. This temporal expression suggests that PDGFD might function as a differentiation “timer” or “amplifier”, ensuring the robust activation of the myogenic transcriptional cascade once proliferation subsides. The less pronounced effect of *PDGFD* overexpression on promoting differentiation could be attributed to the already high differentiation efficiency of C2C12 cells or the existence of tight upstream regulatory checkpoints.

### 4.3. Divergence from the Canonical Pathway: Implications for Context-Dependent PDGFD Signaling

A pivotal and intriguing finding of our study is the apparent dissociation between PDGFD’s function and the sustained activation of the PI3K/Akt pathway in the context of differentiation. The PI3K/Akt pathway is a major downstream effector of PDGFR-β, renowned for promoting cell survival and growth [17,18]. As expected, pharmacological hyper-activation of Akt with SC79 potently inhibited terminal differentiation, consistent with the literature showing that sustained Akt signaling can block myogenic conversion. However, the lack of an additive or synergistic inhibitory effect when combining *PDGFD* knockdown with SC79 treatment is revealing. This non-additivity suggests that the inhibitory effect of PDGFD loss on differentiation is not simply mediated by attenuating the same PI3K/Akt survival signal that, when exogenously maximized, also blocks differentiation.

This observation points to a more complex, context-dependent signaling mechanism. We propose two non-exclusive possibilities: First, *PDGFD* may regulate differentiation through a parallel signaling branch downstream of *PDGFR-β*, such as the MAPK/ERK pathway, which has been shown to have nuanced, stage-specific roles in myogenesis. Second, PDGFD’s nuclear localization observed in our immunofluorescence study (though preliminary) hints at potential non-canonical, intracellular functions, perhaps as a co-regulator of transcription independent of its secreted growth factor activity. This divergence underscores that PDGFD’s role is not monolithic; its pro-survival and pro-differentiation outputs may be uncoupled, allowing it to fulfill sequential functions during myogenesis.

### 4.4. Limitations and Future Perspectives

This study has several limitations that chart the course for future research. First, while we used molecular markers, direct morphological quantification of myotube formation (e.g., fusion index, myotube diameter) would strengthen the differentiation phenotype. Second, the preliminary snRNA-seq data from pigs suggest PDGFD’s co-involvement in adipocyte biology, raising the fascinating question of how it might coordinate or decide between myogenic and adipogenic fates in mesenchymal precursors—an area ripe for exploration. Third, definitively mapping the signaling pathway responsible for PDGFD’s pro-differentiation effect requires further work, such as using pathway-specific inhibitors alongside rescue experiments.

## 5. Conclusions

In conclusion, we have expanded the functional repertoire of *PDGFD* from a classical mesenchymal mitogen to a dual-regulator of skeletal myogenesis. It safeguards the myoblast pool by ensuring survival and subsequently facilitates differentiation through a mechanism that appears distinct from its canonical pro-survival PI3K/Akt pathway. This work not only identifies *PDGFD* as a novel player in muscle biology but also highlights the sophisticated, stage-specific signaling logic employed by growth factors to guide complex developmental processes like myogenesis.

## Figures and Tables

**Table 1:** The primer sequences of PCR.

Primer	Sequence
*PDGFD*-Forward (5′-3′)	ATGCACCGGCTCATCCTCGTCTACA
*PDGFD*-Reverse (5′-3′)	TTATCGAGGTGGTCTTGAGCTGCAGATACAATC

**Table 2:** Interference fragment sequence.

Sequence Number (Markers in the Experiment)	S	AS
*PDGFD*-MUS-963 (SI-1)	CCGUGGAAGAUCUACUUAATT	UUAAGUAGAUUCCACGGTT
*PDGFD*-MUS-9-423 (SI-2)	GGAGAGAUGAGAGCAAUCATT	UGAUUGCUCUCAUCUCUCCTT
*PDGFD*-MUS-9-1092 (SI-3)	GGCUCAAUGAUGAUGUCAATT	UUGACAUCAUCAUUGAGCCTT
*PDGFD*-MUS-9716 (SI-4)	CCUCCAAGGAUAACGUCAATT	UUGACGUUAUCCUUGGAGGTT
Nagative control	UUCUCCGAACGUGUCACGUTT	ACGUGACACGUUCGGAGAATT

Note: S represents the sense strand, while AS represents the antisense strand. SI-2 was experimentally found to have no interference efficiency, so it is not considered in subsequent presentations.

**Table 3:** The primer sequences of RT-qPCR.

Gene	Forward (5′-3′)	Reverse (5′-3′)
Mus-*PDGFD* (*NC_000075.7*)	TGAGAGCAATCACCTCACAGAC	CAGAAGCAGGTTCCTTGGGT
Mus-*MYOD* (*NC_000073.7*)	TCCAACTGCTCTGATGGCATGATG	ACTGTAGTAGGCGGTGTCGTAGC
Mus-*MYOG* (*NC_000067.7*)	GAGACATCCCCCTATTTCTACCA	GCTCAGTCCGCTCATAGCC
Mus-*MEF2C* (*NC_000079.7*)	AAGCCTCAGCATCAAGTCAGAACC	GCGTGGTGTGTTGTGGGTATCTC
Mus-*β-actin* (*NC_000071.7*)	GTGACGTTGACATCCGTAAAGA	GCCGGACTCATCGTACTCC

## Data Availability

The original contributions presented in this study are included in the article/Supplementary Materials. Further inquiries can be directed to the corresponding authors.

## Supplementary Materials

The following supporting information can be downloaded at https://www.mdpi.com/article/10.3390/cimb48030322/s1.
