# Supplementary figures and images for: PDGFD: A Dual-Function Regulator That Maintains Myoblast Pool and Fuels Myogenic Differentiation
Source: Curr Issues Mol Biol. 2026 Mar 18;48(3):322. doi: 10.3390/cimb48030322 (PMC13025337; doi:10.3390/cimb48030322)

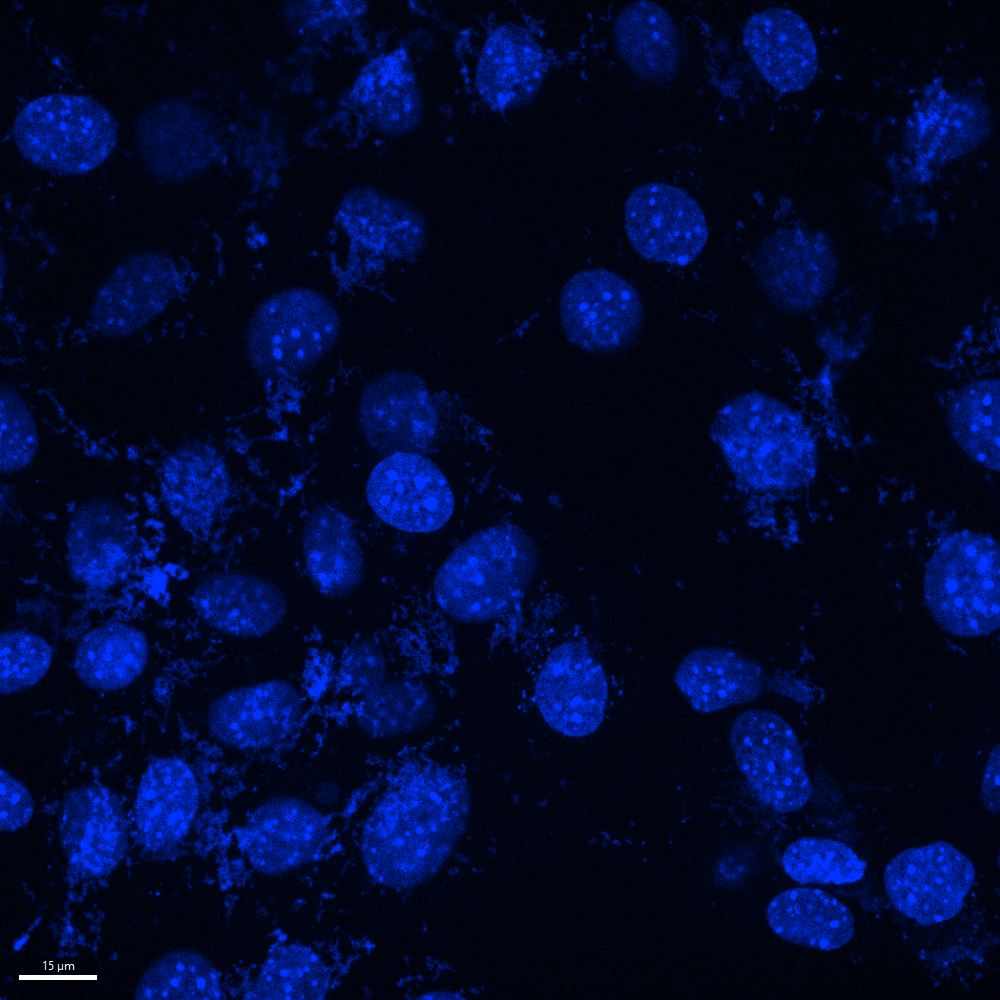

Supplement: Supplementary file 1 [file cimb-48-00322-s001.zip › S1/D0/DAPI.tif]

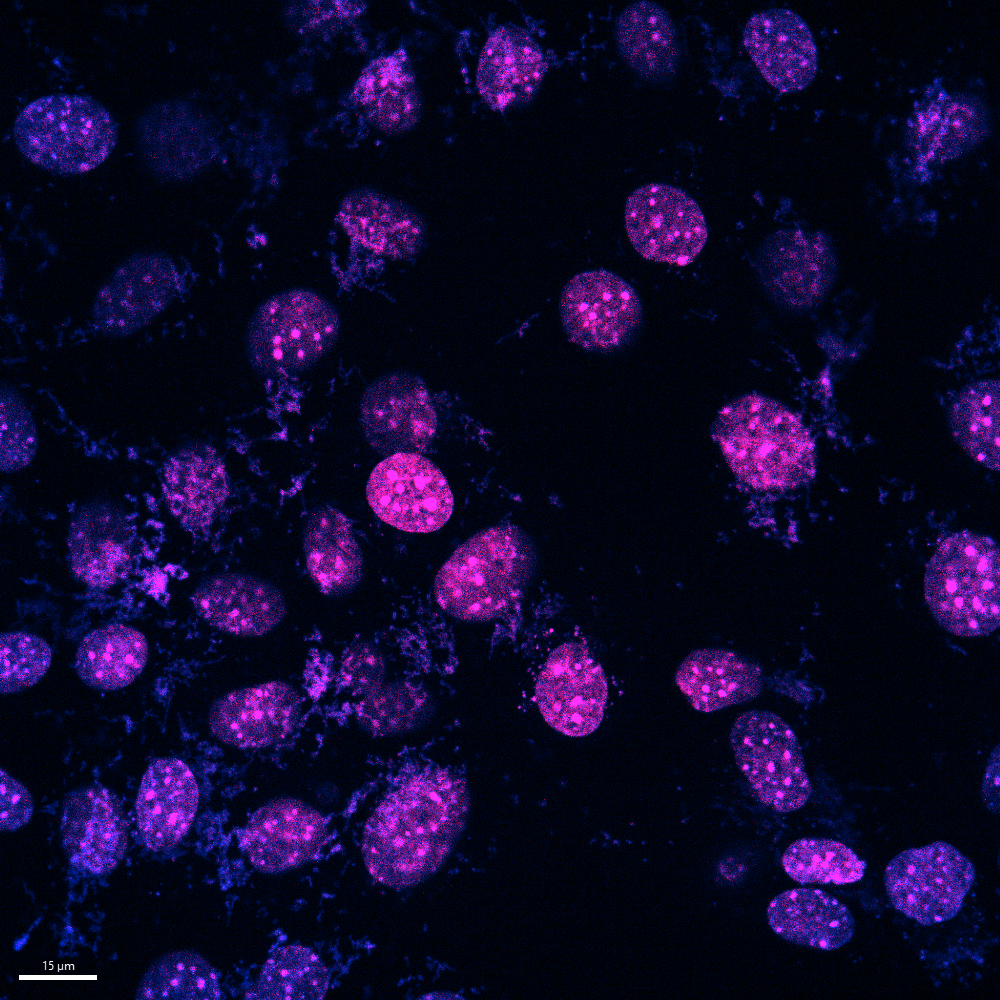

Supplement: Supplementary file 1 [file cimb-48-00322-s001.zip › S1/D0/Merge.tif]

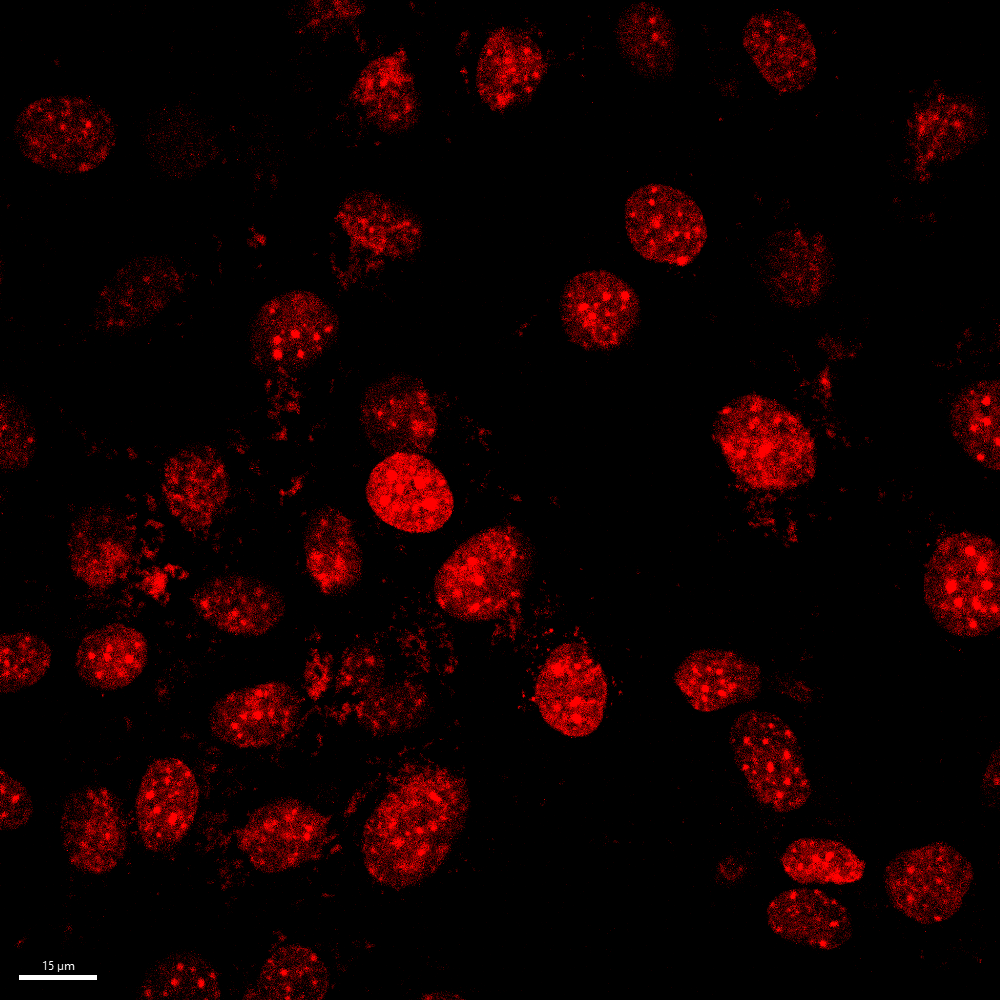

Supplement: Supplementary file 1 [file cimb-48-00322-s001.zip › S1/D0/PDGFD.tif]

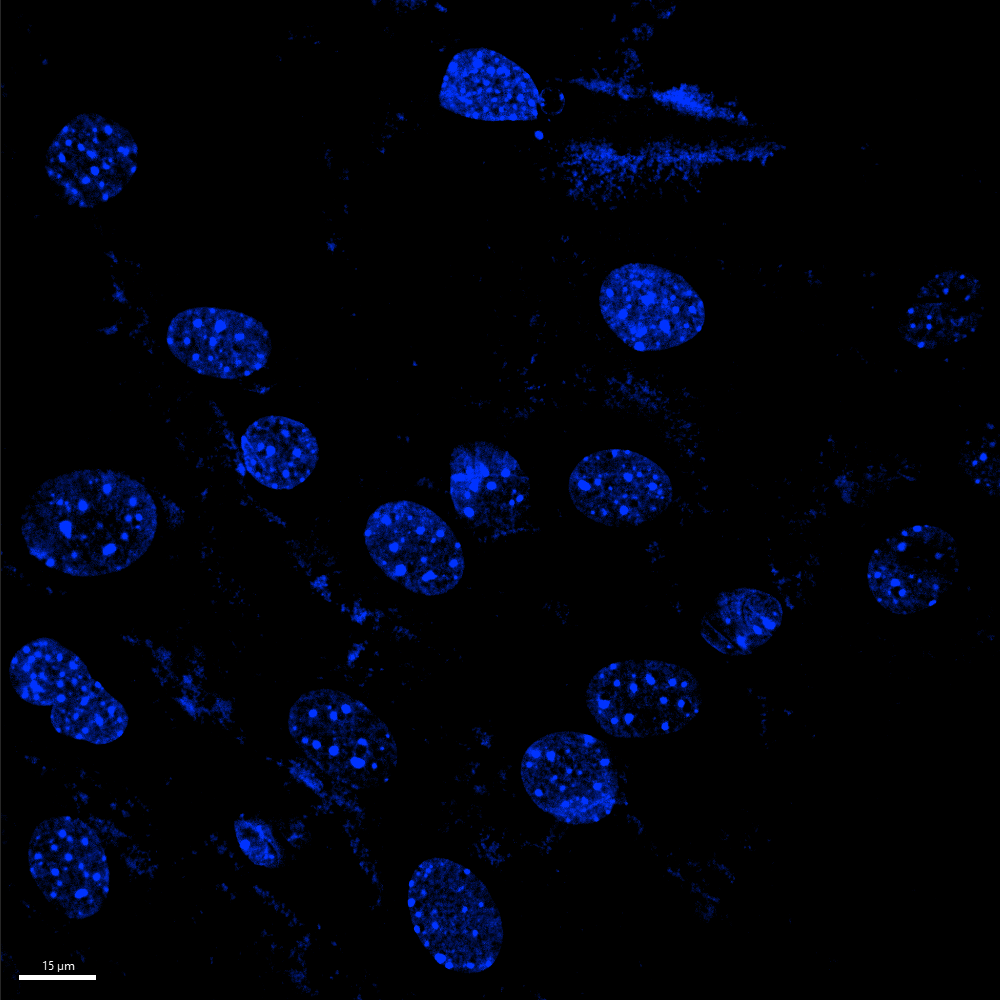

Supplement: Supplementary file 1 [file cimb-48-00322-s001.zip › S1/D5/DAPI.tif]

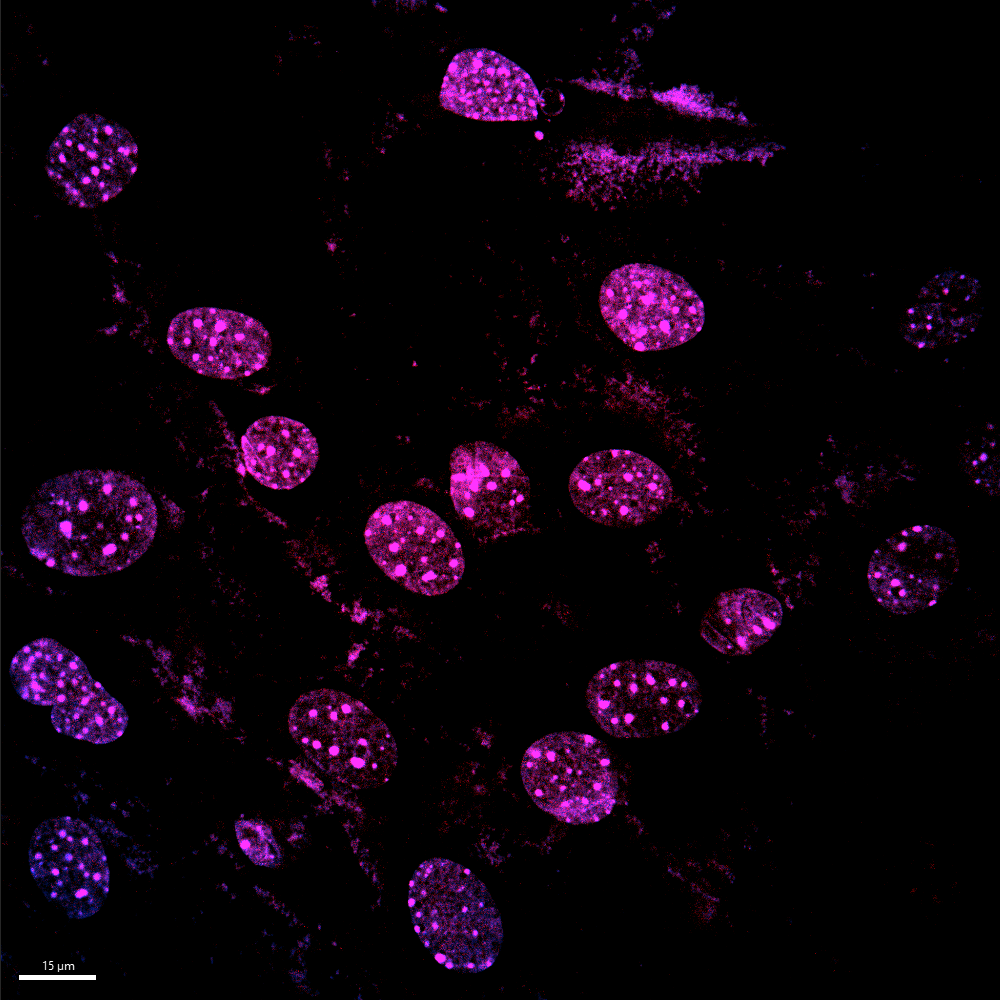

Supplement: Supplementary file 1 [file cimb-48-00322-s001.zip › S1/D5/Merge.tif]

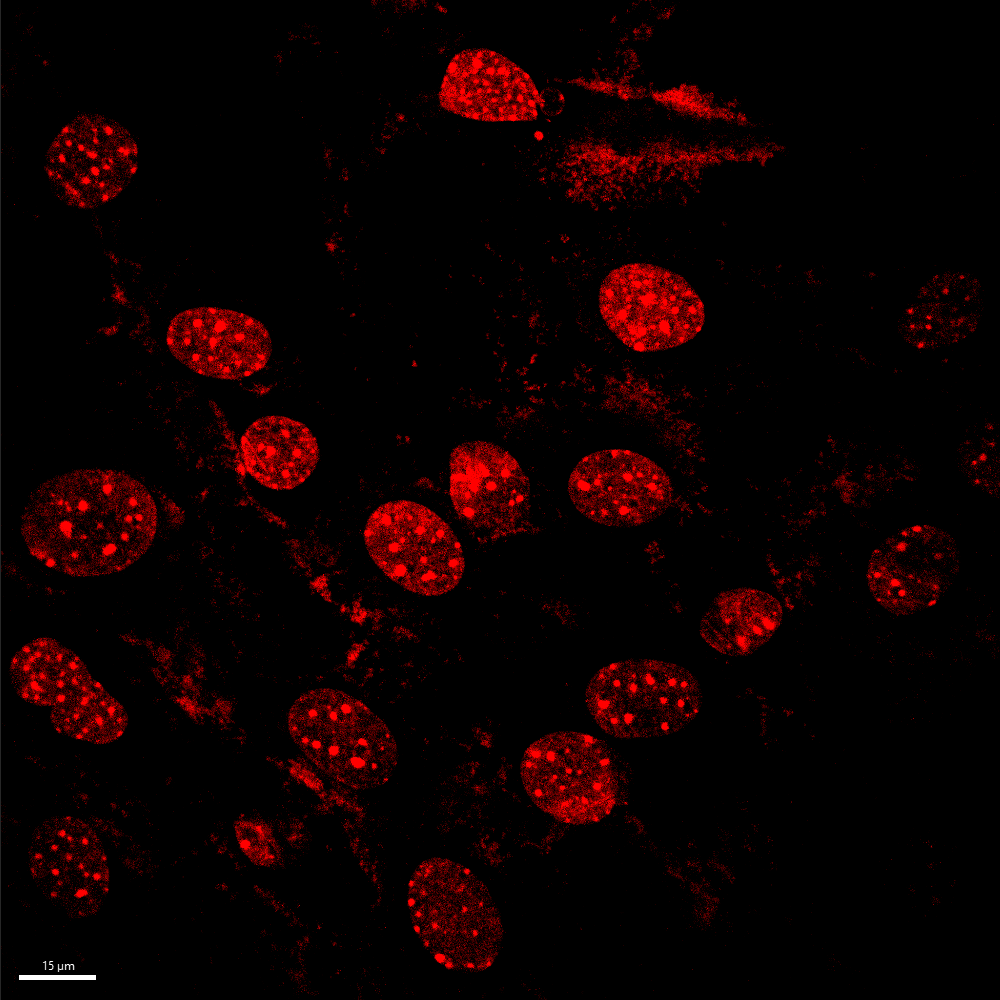

Supplement: Supplementary file 1 [file cimb-48-00322-s001.zip › S1/D5/PDGFD.tif]

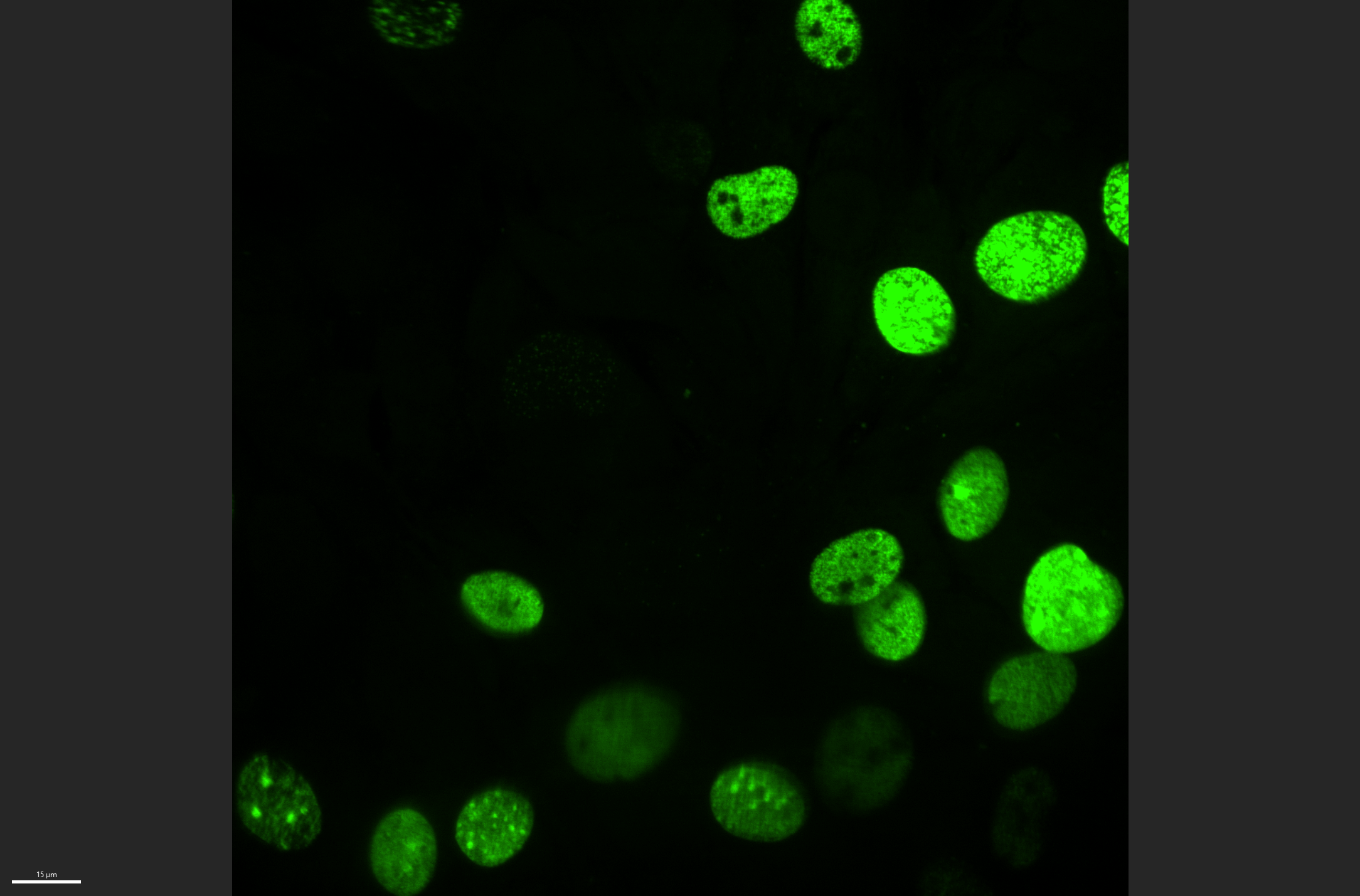

Supplement: Supplementary file 1 [file cimb-48-00322-s001.zip › S2/NC/EdU.tif]

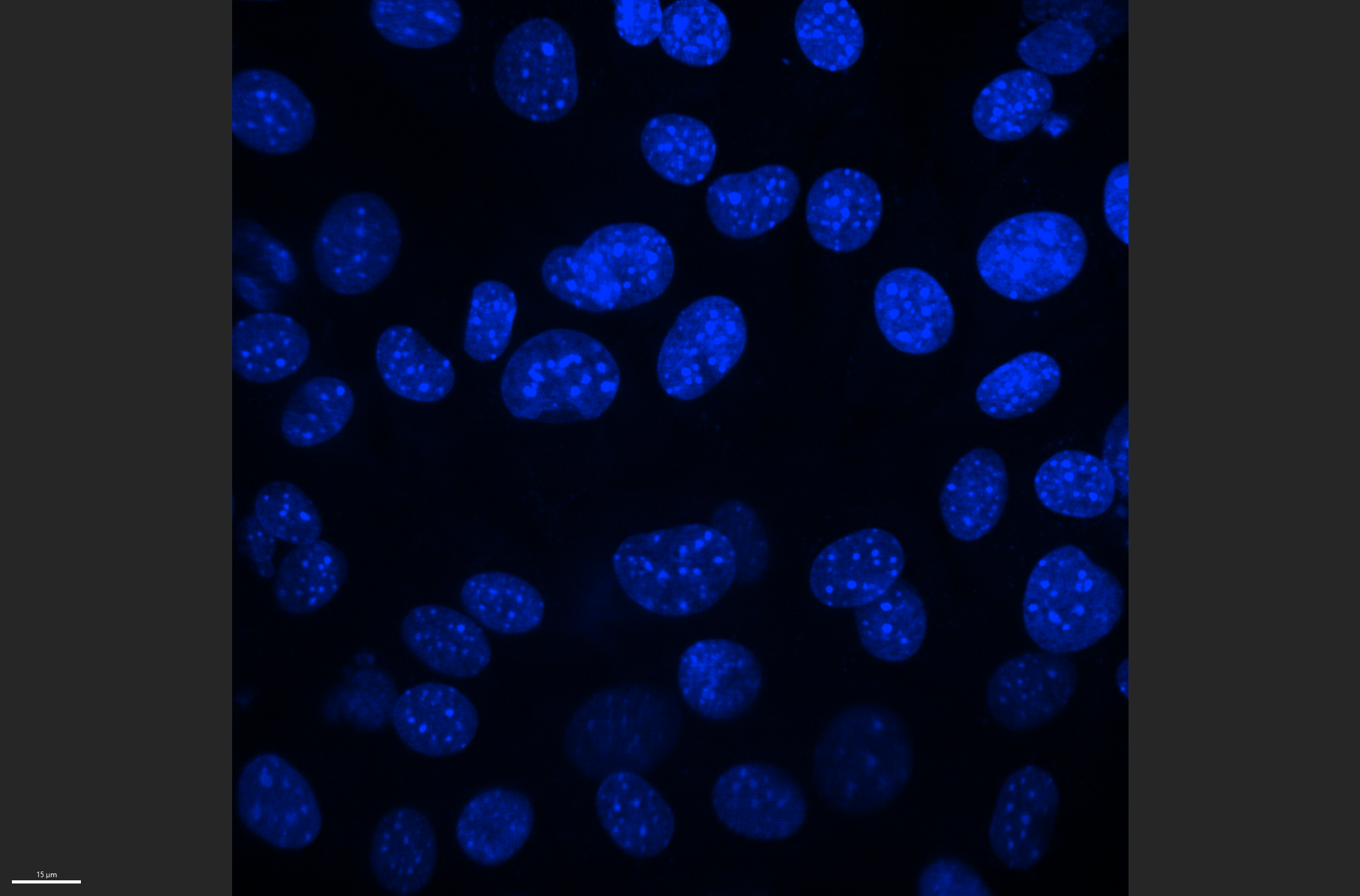

Supplement: Supplementary file 1 [file cimb-48-00322-s001.zip › S2/NC/Hoechst.tif]

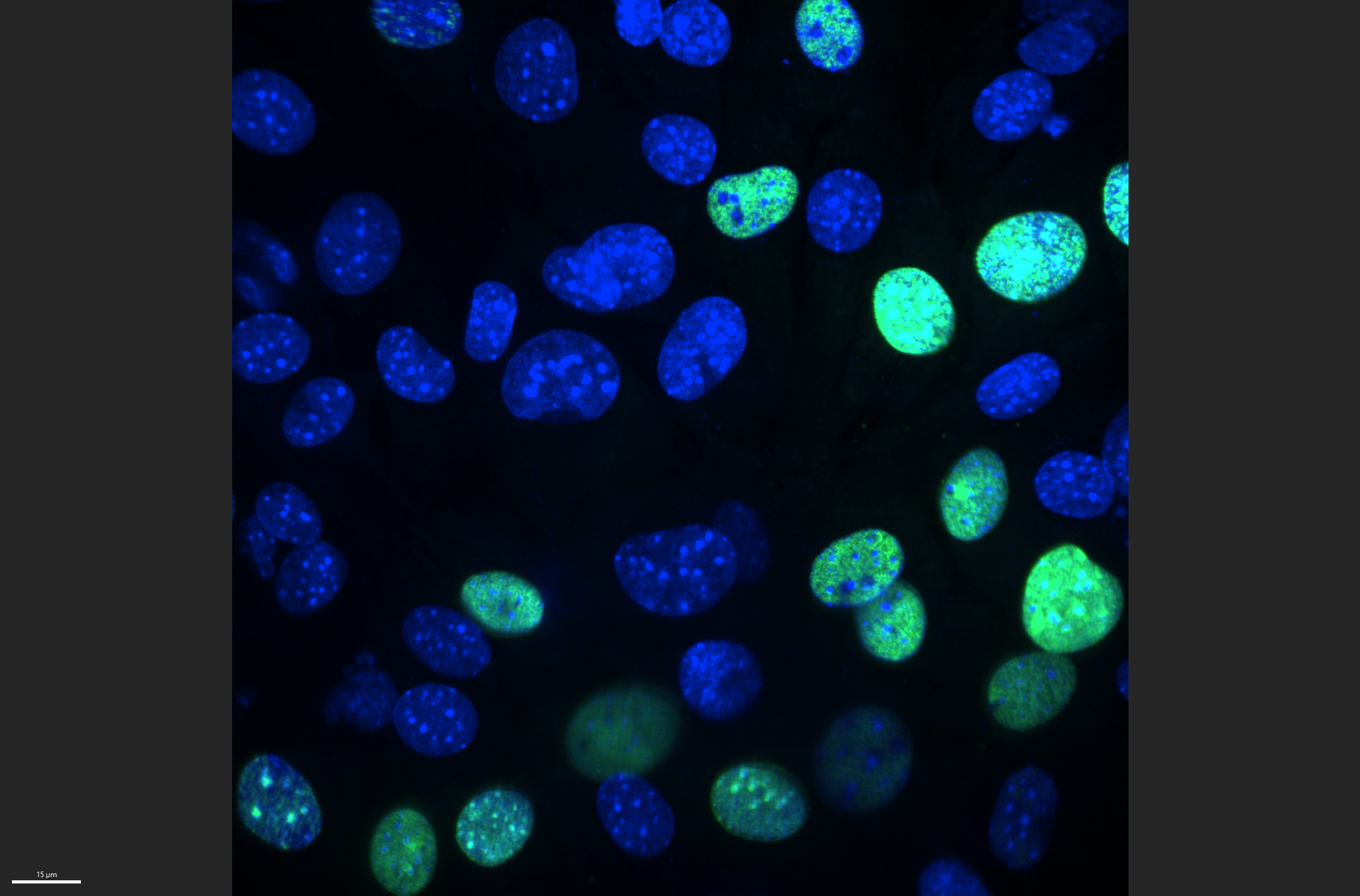

Supplement: Supplementary file 1 [file cimb-48-00322-s001.zip › S2/NC/Merge.tif]

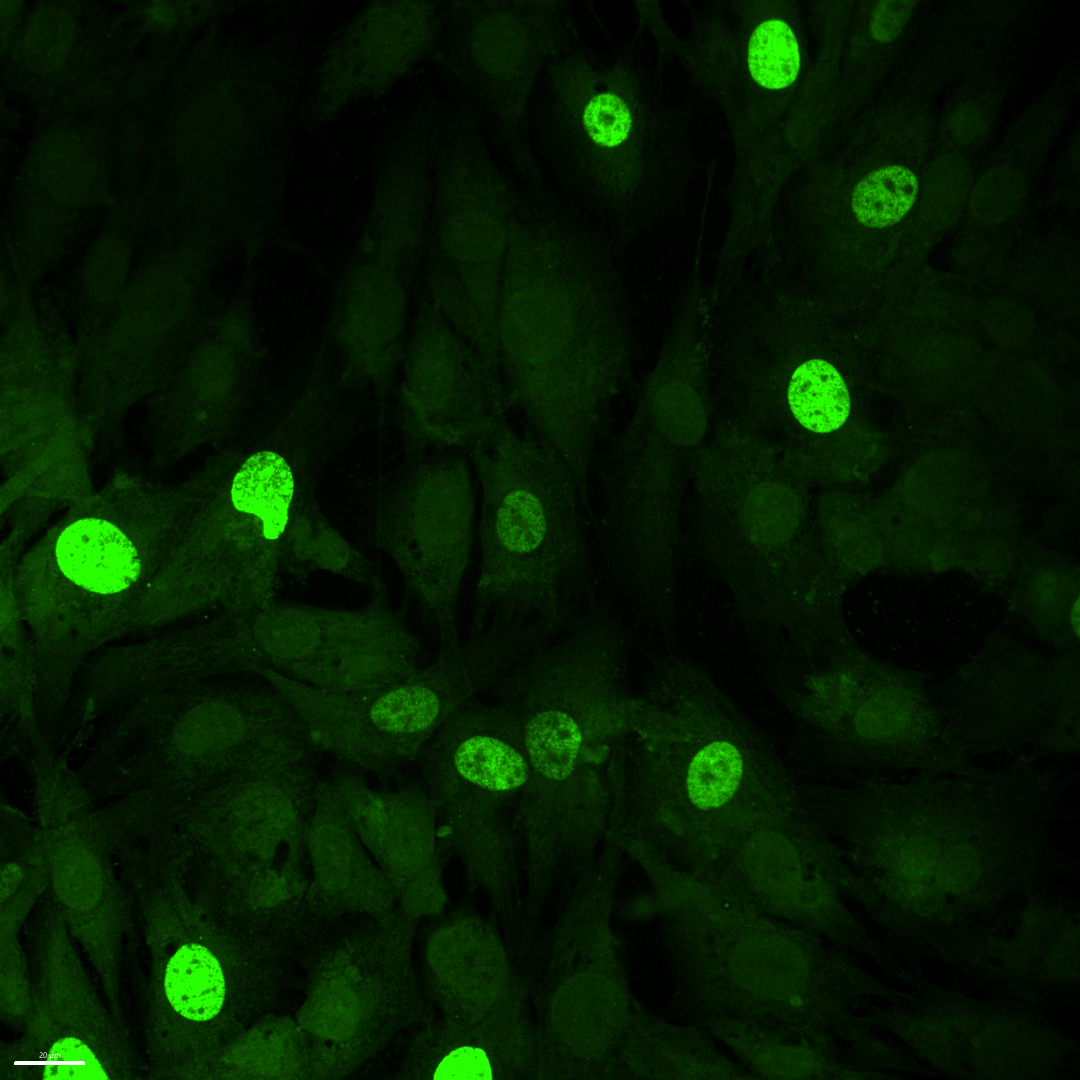

Supplement: Supplementary file 1 [file cimb-48-00322-s001.zip › S2/pEGFP-N1/EdU.tif]

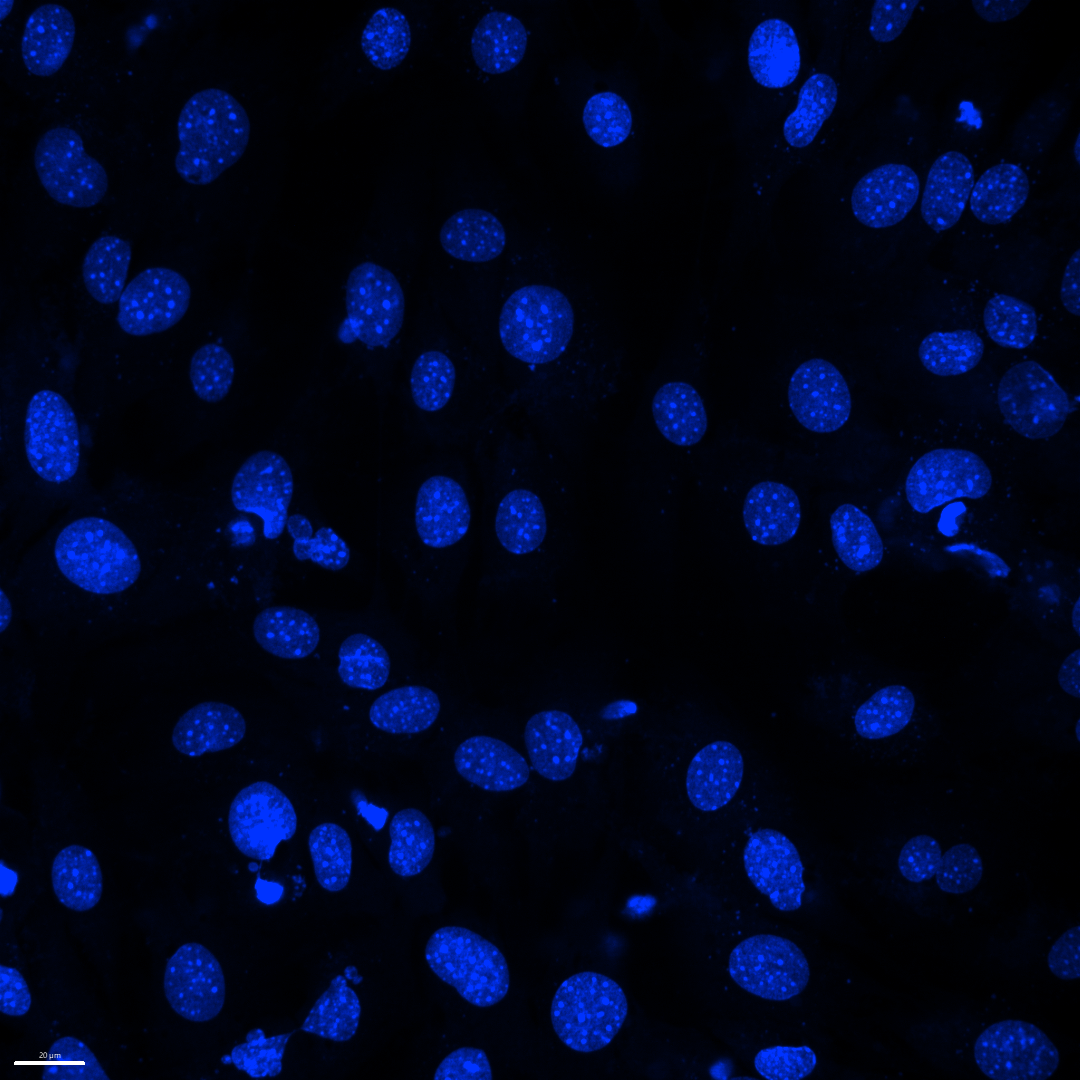

Supplement: Supplementary file 1 [file cimb-48-00322-s001.zip › S2/pEGFP-N1/Hoechst.tif]

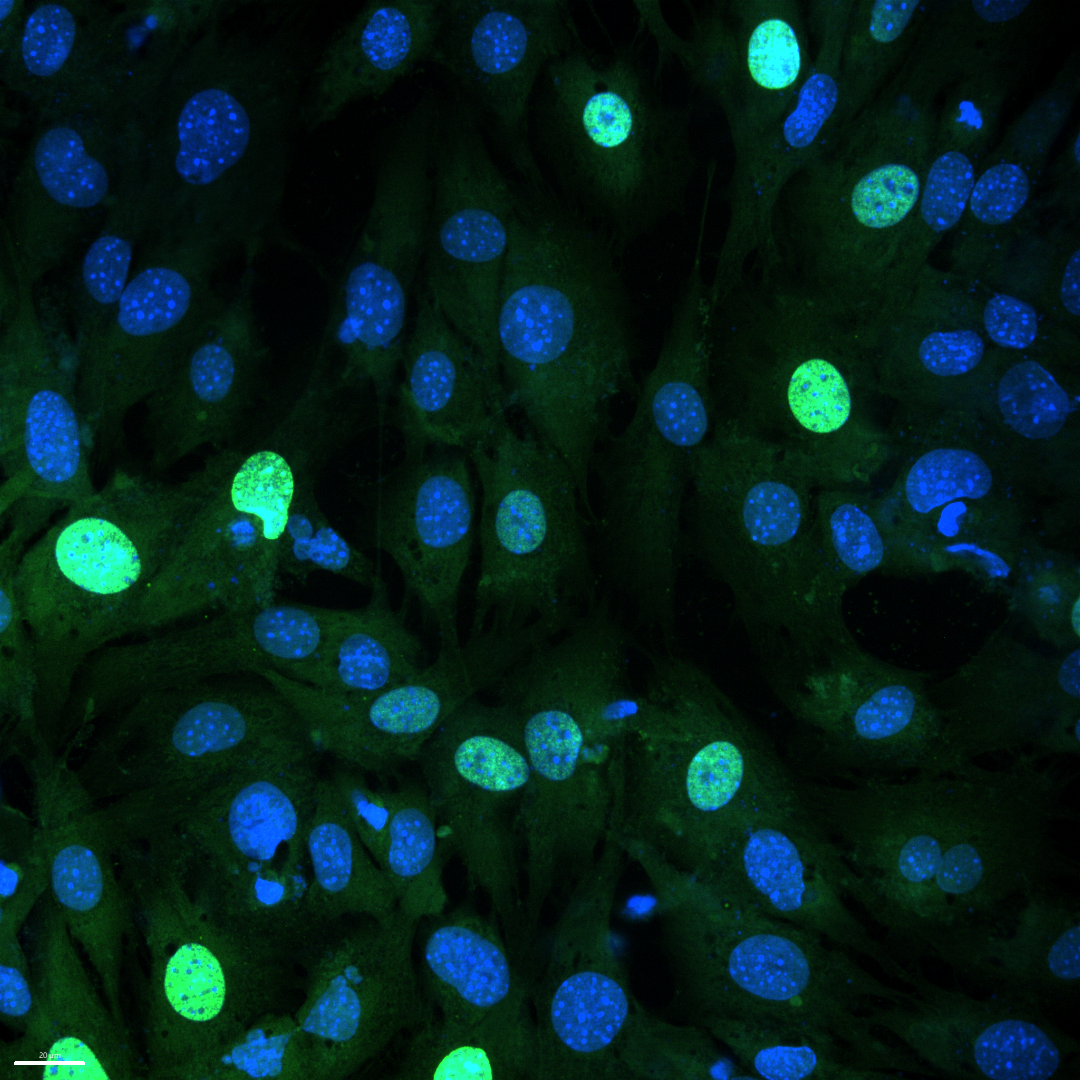

Supplement: Supplementary file 1 [file cimb-48-00322-s001.zip › S2/pEGFP-N1/Merge.tif]

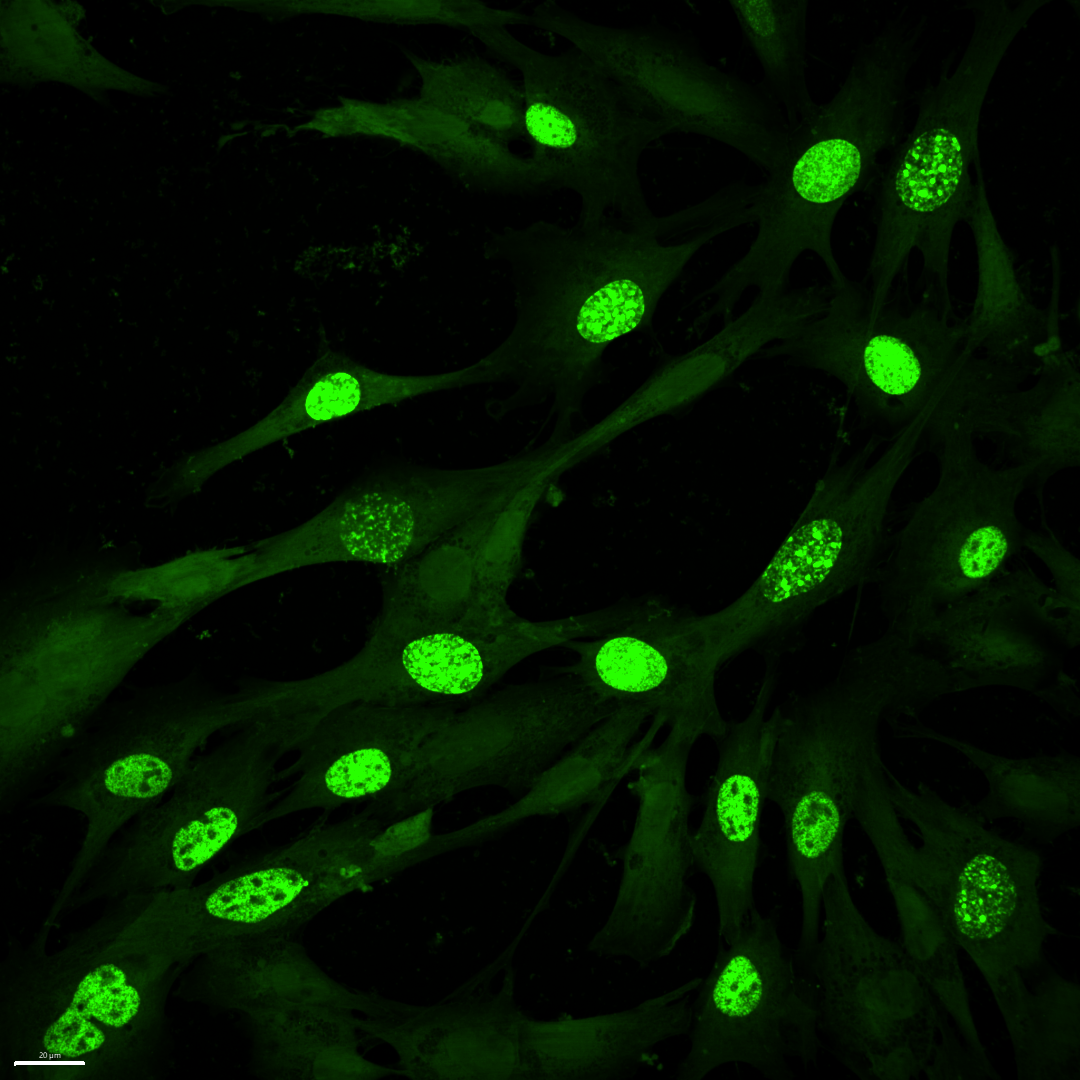

Supplement: Supplementary file 1 [file cimb-48-00322-s001.zip › S2/pEGFP-N1-PDGFD/EdU.tif]

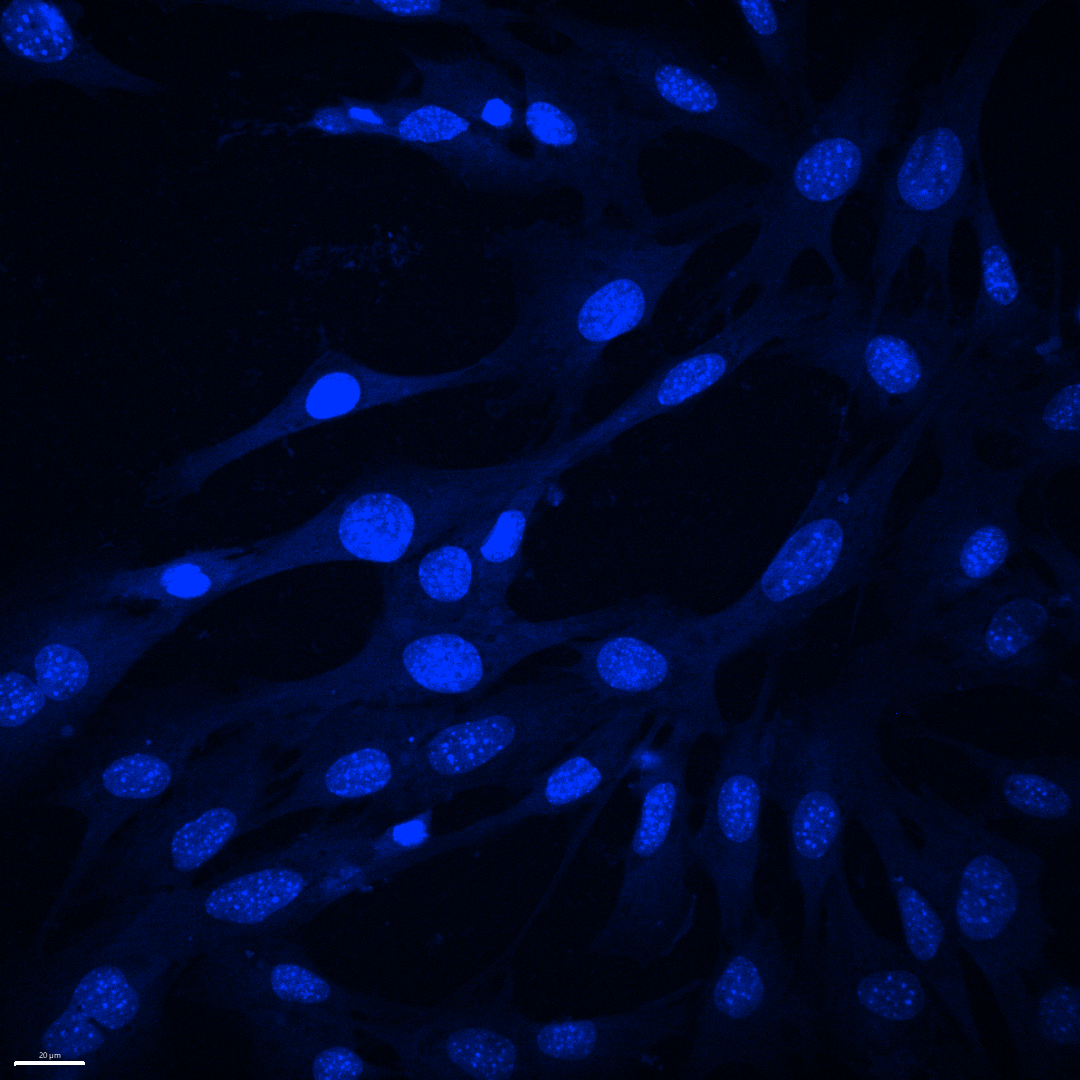

Supplement: Supplementary file 1 [file cimb-48-00322-s001.zip › S2/pEGFP-N1-PDGFD/Hoechst.tif]

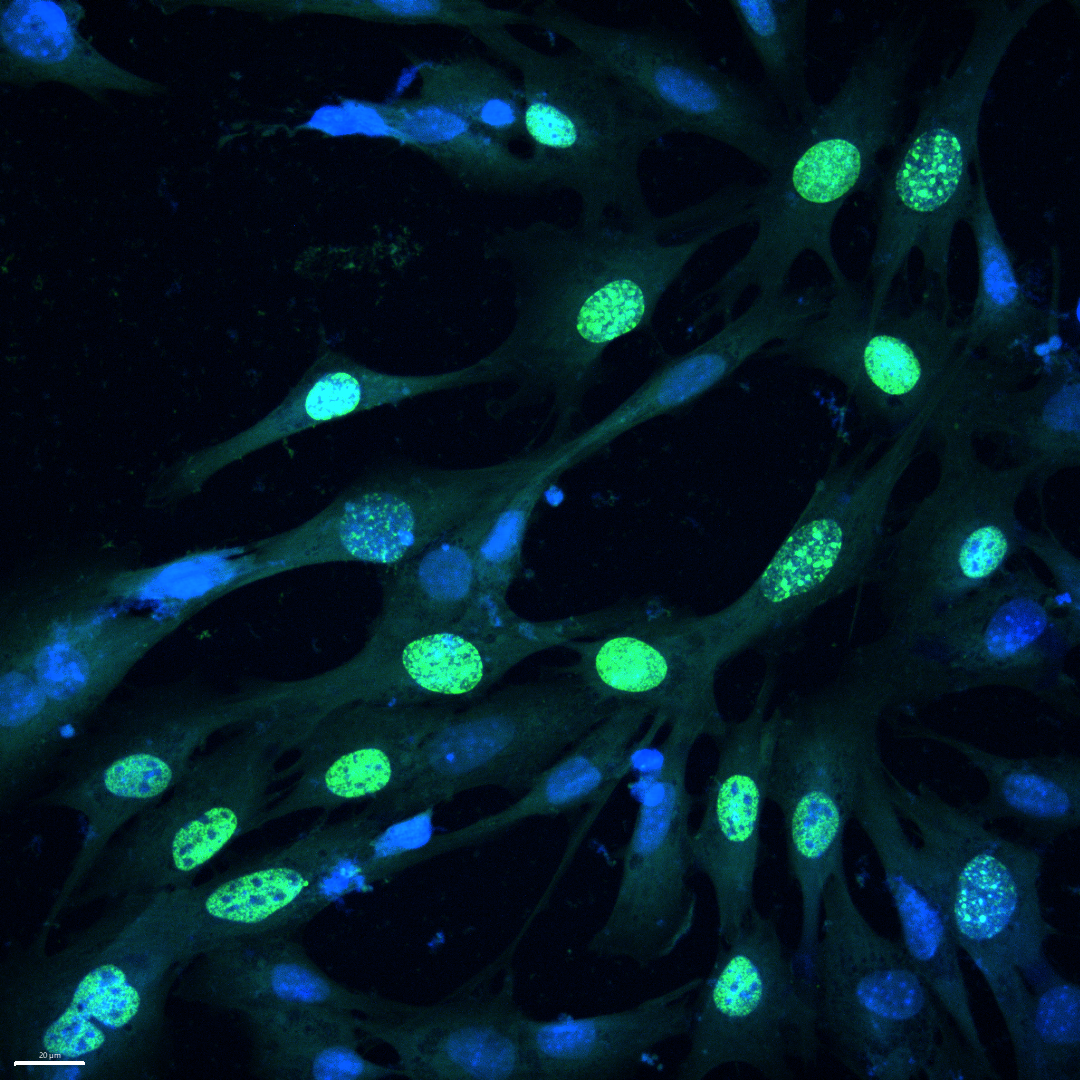

Supplement: Supplementary file 1 [file cimb-48-00322-s001.zip › S2/pEGFP-N1-PDGFD/Merge.tif]

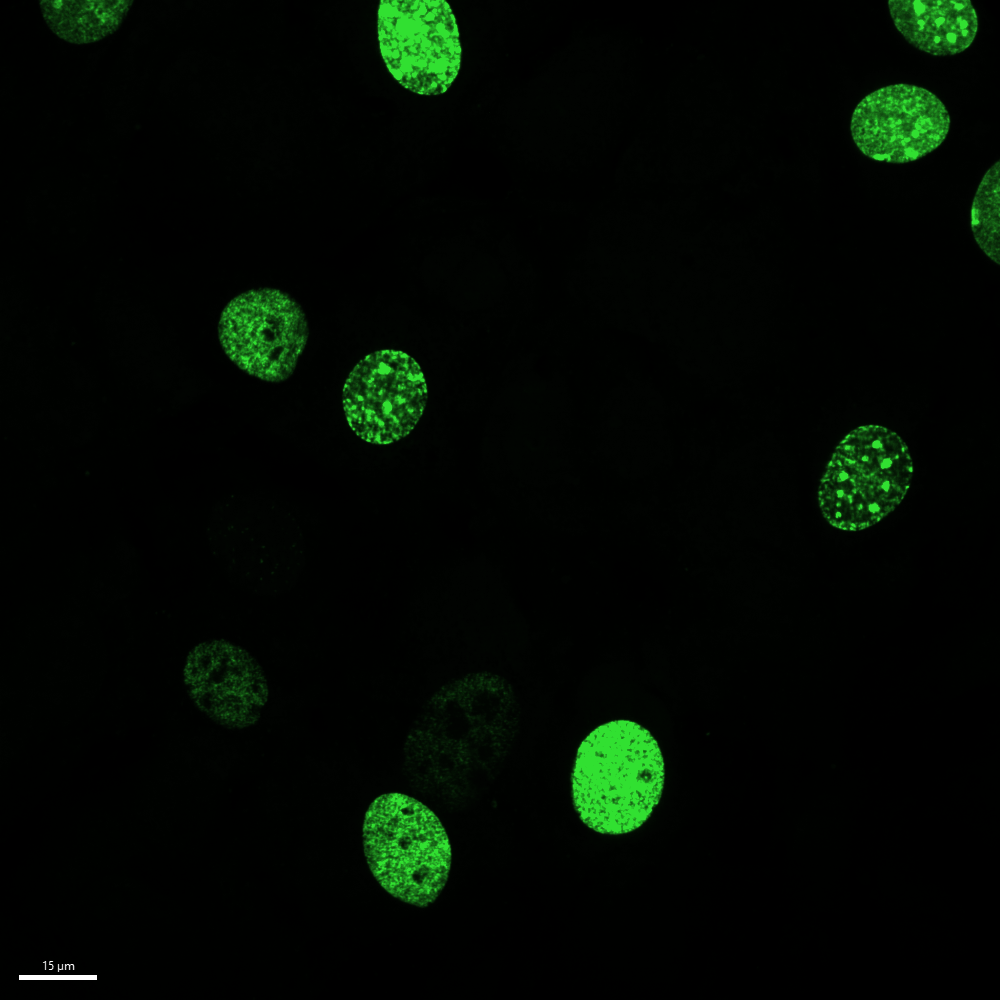

Supplement: Supplementary file 1 [file cimb-48-00322-s001.zip › S2/SI-PDGFD/EdU.tif]

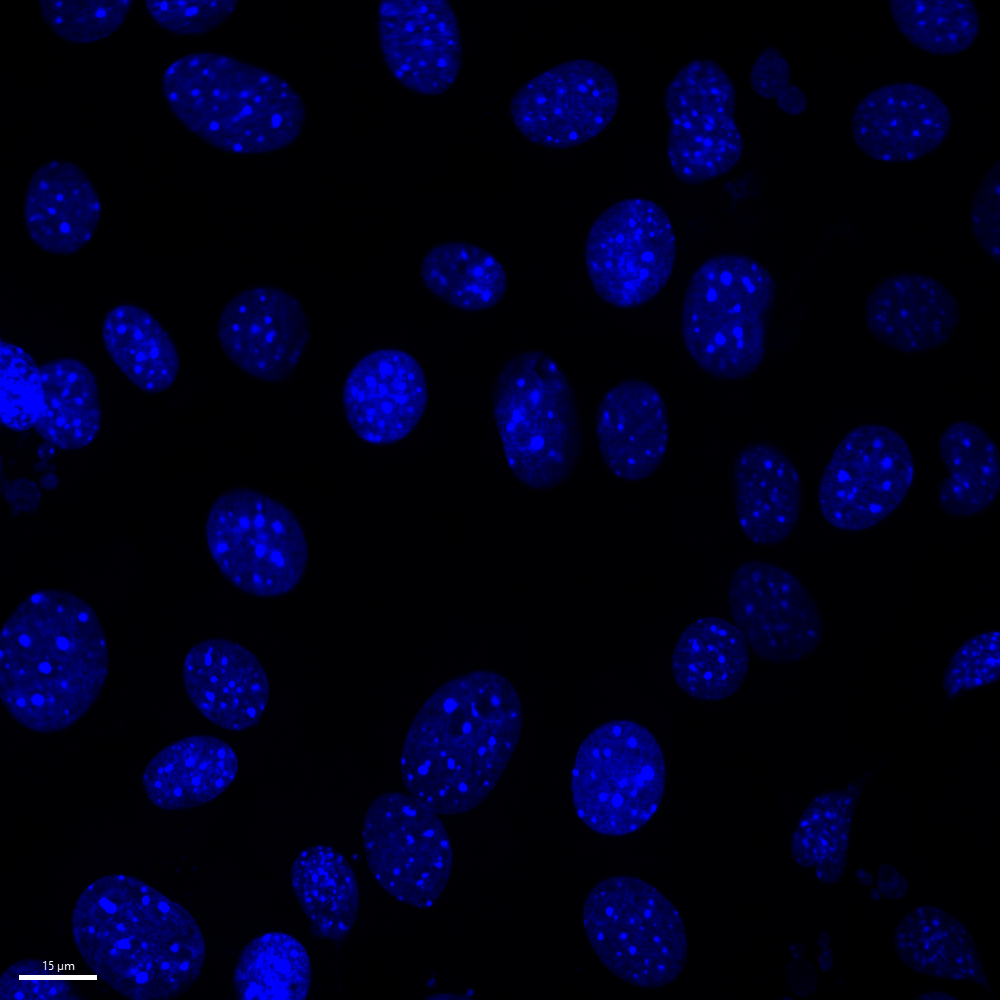

Supplement: Supplementary file 1 [file cimb-48-00322-s001.zip › S2/SI-PDGFD/Hoechst.tif]

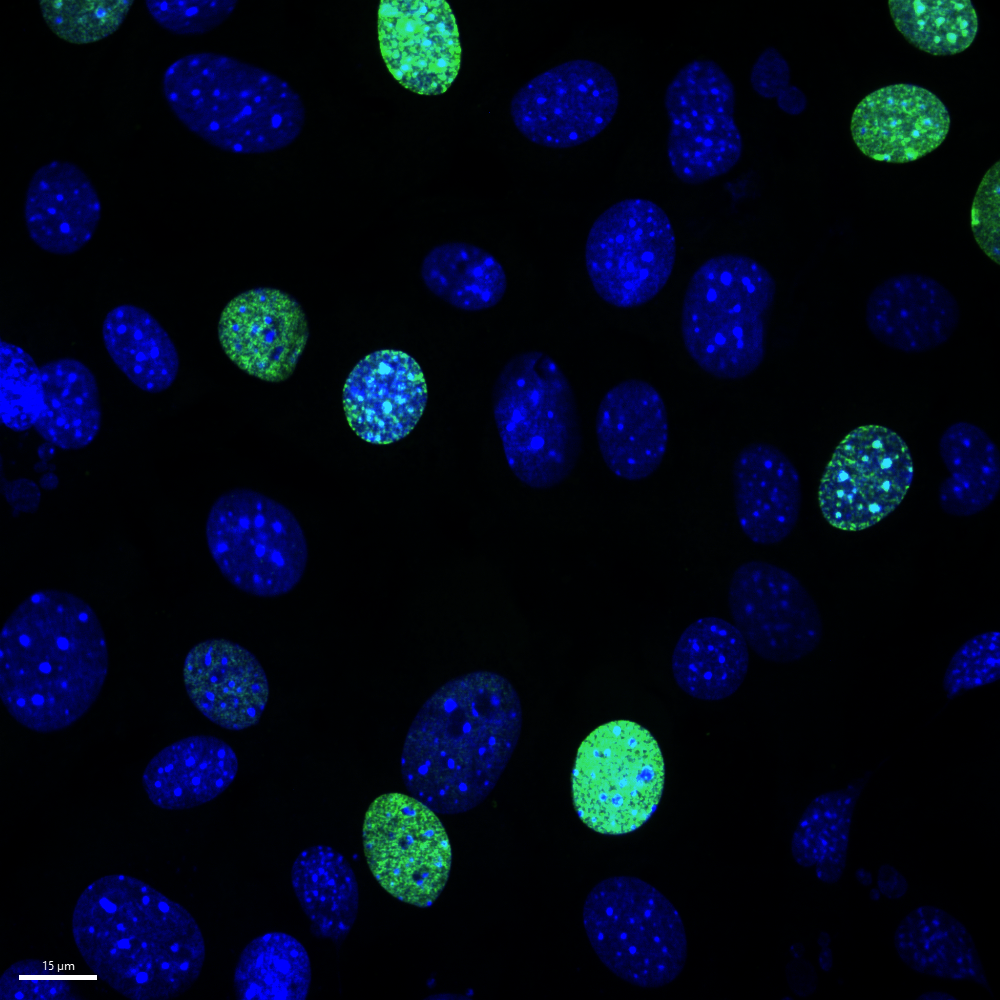

Supplement: Supplementary file 1 [file cimb-48-00322-s001.zip › S2/SI-PDGFD/Merge.tif]

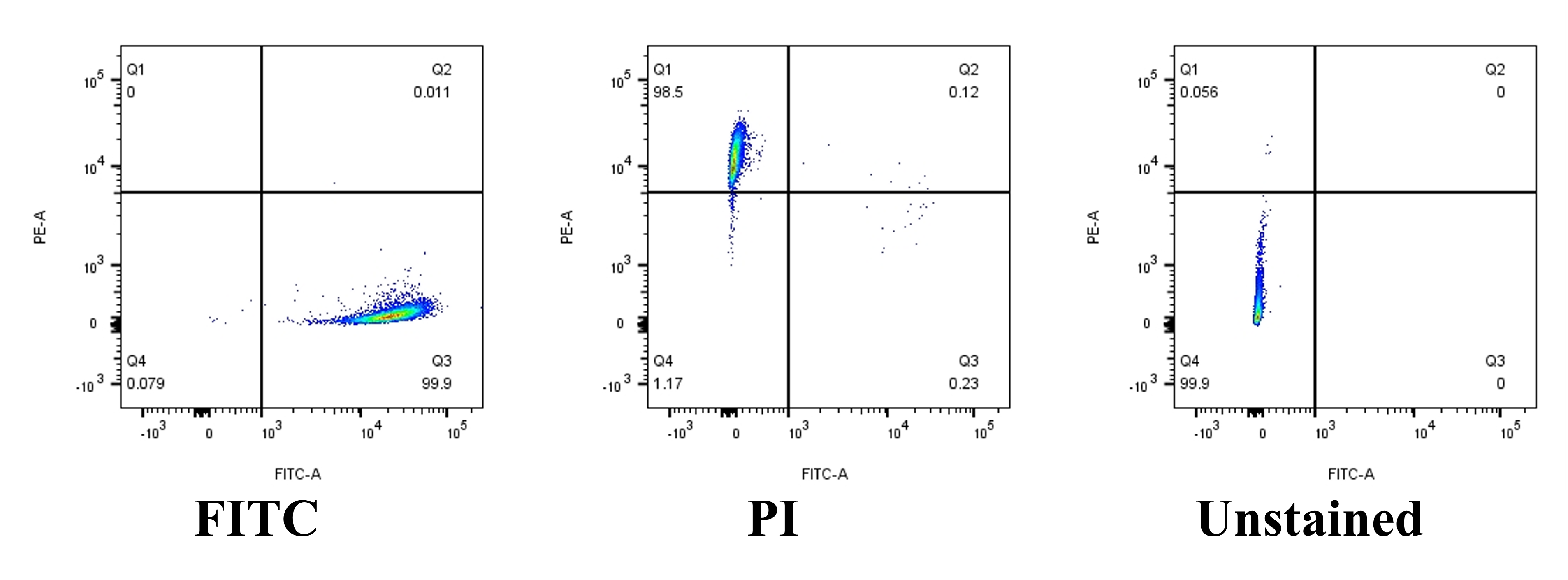

Supplement: Supplementary file 1 [file cimb-48-00322-s001.zip › S3.png]
